# Topology‐Dependent Coke Formation in the Catalytic Pyrolysis of Phenol Over HFAU and HZSM‐5 Zeolites

**DOI:** 10.1002/anie.202523882

**Published:** 2026-04-09

**Authors:** Jörg W. A. Fischer, Allen Puente‐Urbina, Zeyou Pan, Mikhail Agrachev, Patrick Hemberger, Gunnar Jeschke, Jeroen A. van Bokhoven

**Affiliations:** ^1^ Institute for Molecular Physical Science ETH Zurich Zurich Switzerland; ^2^ Institute for Catalysis Hokkaido University Sapporo Japan; ^3^ Institute for Chemical and Bioengineering ETH Zurich Zurich Switzerland; ^4^ School of Chemistry Costa Rica Institute of Technology Cartago Costa Rica; ^5^ Laboratory for Synchrotron Radiation and Femtochemistry Paul Scherrer Institute Villigen Switzerland; ^6^ Center for Energy and Environmental Sciences Paul Scherrer Institute Villigen Switzerland

**Keywords:** coke formation, DFT, heterogeneous catalysis, lignin pyrolysis, operando EPR, zeolites

## Abstract

Catalytic pyrolysis of lignin, the most abundant natural aromatic polymer, offers a route to obtain value‐added products with a low carbon footprint. In such a process, the lignin structure undergoes decomposition through an intricate network of reaction routes. Despite the use of model compounds to gain insights into the decomposition pathways, the formation mechanism of coke and its role in critically affecting catalyst performance remain poorly understood. Herein, we use *operando* electron paramagnetic resonance (EPR) spectroscopy together with ex situ pulsed EPR experiments and density functional theory (DFT) calculations to understand coke formation in catalytic pyrolysis of phenol over HFAU and HZSM‐5 zeolites. Our results pinpoint that coke formation is heavily influenced by zeolite topology. The large cages in HFAU facilitate the initial formation of linear configurations that grow to extended structures, whereas the narrower channels in HZSM‐5 promote the formation of more linear structures. These results provide comprehensive mechanistic insights into coke formation and growth that are relevant for the development of lignin valorization strategies and for the general phenomenon of coke formation in zeolites and beyond.

## Graphical abstract

The coke formation and speciation during the catalytic pyrolysis of lignin over different zeolite topologies were studied with a combination of *operando* EPR spectroscopy and ex situ pulsed techniques aided with DFT calculations. *Operando* EPR data showed that the coke formation in HFAU is fast compared to HZSM‐5. While ex situ measurement revealed that coke deposits in HZSM‐5 zeolites adopt a more linear structure than in HFAU.

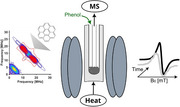

## 1 Introduction

A fundamental challenge in replacing fossil resources in the chemical and energy sectors is the need to adopt sustainable carbon‐based feedstocks to produce the chemicals, fuels, and materials central to modern society [1, 2]. A compelling approach to addressing this need is the utilization of lignocellulosic biomass [3]. It is primarily composed of three biopolymers: cellulose, hemicellulose, and lignin. Lignin is an irregular polymer, consisting primarily of various phenolic monomers connected by diverse linkages, whose type and frequency depend on the origin of the biomass [4, 5, 6, 7]. Catalytic pyrolysis of lignin over zeolite‐based catalysts (e.g., HFAU and HZSM‐5) offers a promising route to high‐value aromatics, but it remains technically challenging due to lignin's complex structure and the broad distribution of resulting products [8, 9, 10]. Simplified molecular studies using model compounds provide critical insights into the fundamental decomposition chemistry of lignin, which is essential for optimizing biomass conversion processes and catalyst development. As a major lignin pyrolysis product that also features a structural motif common in lignin, phenol serves as an effective model compound for studying thermal decomposition pathways [11, 12, 13, 14, 15].

**SCHEME 1:**
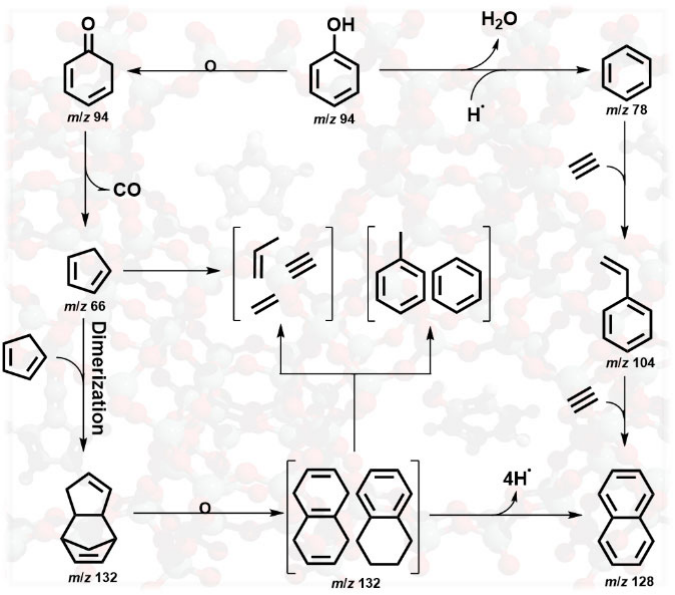
The proposed reaction pathways of phenol in HZSM‐5‐catalyzed pyrolysis, simplified based on Pan et al. [12]. The reactions happen on the catalyst; the hydrogen radical represents the proton in the catalyst.

Our previous report [12] employing imaging photoelectron photoion coincidence (*i*PEPICO) spectroscopy with synchrotron radiation shows that phenol (*m/z* 94) decomposition under HZSM‐5‐catalyzed pyrolysis proceeds via two main pathways (Scheme 1): (i) Phenol can decompose to cyclopentadiene via 4‐cyclohexadienone (C_6_H_6_O, *m/z* 94), which subsequently undergoes ring opening producing small olefins such as acetylene (C_2_H_2_, *m/z* 26). Cyclopentadiene readily dimerizes to dicyclopentadiene (C_10_H_12_, *m/z* 132) at high concentrations; the dimer can then either decompose to toluene and benzene or rearrange and dehydrogenate to form naphthalene (C_10_H_8_, *m/z* 128) via tetrahydronaphthalene intermediates (C_10_H_12_, *m/z* 132). (ii) Alternatively, phenol may undergo dehydroxylation to yield benzene on the Brønsted acid sites of HZSM‐5. Benzene can then form naphthalene through the hydrogen‐abstraction/acetylene‐addition (HACA) mechanism in the presence of acetylene. Both pathways can lead to the formation of naphthalene, a key coke precursor that contributes to catalyst deactivation.

Understanding the reaction mechanisms underlying coking is crucial for developing effective catalysts that mitigate deactivation and for establishing effective and efficient reactivation protocols, thereby improving overall process performance [10, 16]. It has been shown that coke deposits in HZSM‐5 (MFI topology) correlate with aluminum‐rich regions, based on combined ^27^Al NMR and atom probe tomography (APT) [17]. Further studies reveal that in HZSM‐5, coke preferentially accumulates along the straight 10‐membered‐ring channels, particularly at the intersections with the sinusoidal 10‐membered‐ring pores. Larger pore sizes can enhance reaction rates by improving molecular diffusion; however, they may also accelerate catalyst deactivation by promoting more extensive coke formation [18, 19]. When the pore architecture shifts from an intersecting channel system to a cage‐based framework, the preferred coke location changes accordingly. In SSZ‐13 (CHA topology), owing to its cage‐type structure, coke is confined within the cages and protrudes through the 8‐membered‐ring windows [20].

Although zeolite deactivation by coke in lignin‐related catalytic pyrolysis has been extensively reported [13, 14, 19], detailed insights into coke formation remain limited. Electron paramagnetic resonance (EPR) spectroscopy under reaction conditions offers a unique, non‐destructive approach to elucidate the underlying processes related to coke formation [10, 16, 21]. EPR enables quantitative analysis of radical coke species, estimated to be 1000–2400 carbon atoms per spin of total coke content, while providing information about the condensation degree of these deposits due to changes in the spectral line shape from continuous wave (CW) experiments [22]. The radical fraction of deposited coke has been considered to represent the overall coke deposits, based on the linear relationship between radical concentration and total coke content, as well as the agreement between ^1^ ^3^C/^1^H ratios obtained from HYSCORE measurements and those determined by elemental analysis from coal samples [23, 24]. A further advantage of EPR is its independence of the optical properties of the sample. In the ongoing reaction, the sample turns black due to the formation of coke, which imposes difficulties for optical and vibrational spectroscopies [25].

In this study, we develop a combined approach of *operando* and ex situ EPR spectroscopy aided by density functional theory (DFT) calculations to reveal the coke structures on zeolite catalysts. To understand the mechanistic influence of the zeolite framework on coking, the unidirectional pore structure of HZSM‐5 (channel diameter ≈ 5.5 Å) was contrasted with the three‐dimensional supercage configuration of HFAU (window opening ≈ 7.4 Å) in catalytic pyrolysis of phenol at 753 K [14, 26]. HZSM‐5 with Si/Al = 25 and HFAU with Si/Al = 2.6 are applied for comparison, as they have similar catalytic activity in transforming phenol, which is produced during guaiacol catalytic pyrolysis, based on our previous studies [12, 14].

## 2 Results and Discussion

**FIGURE 1:**
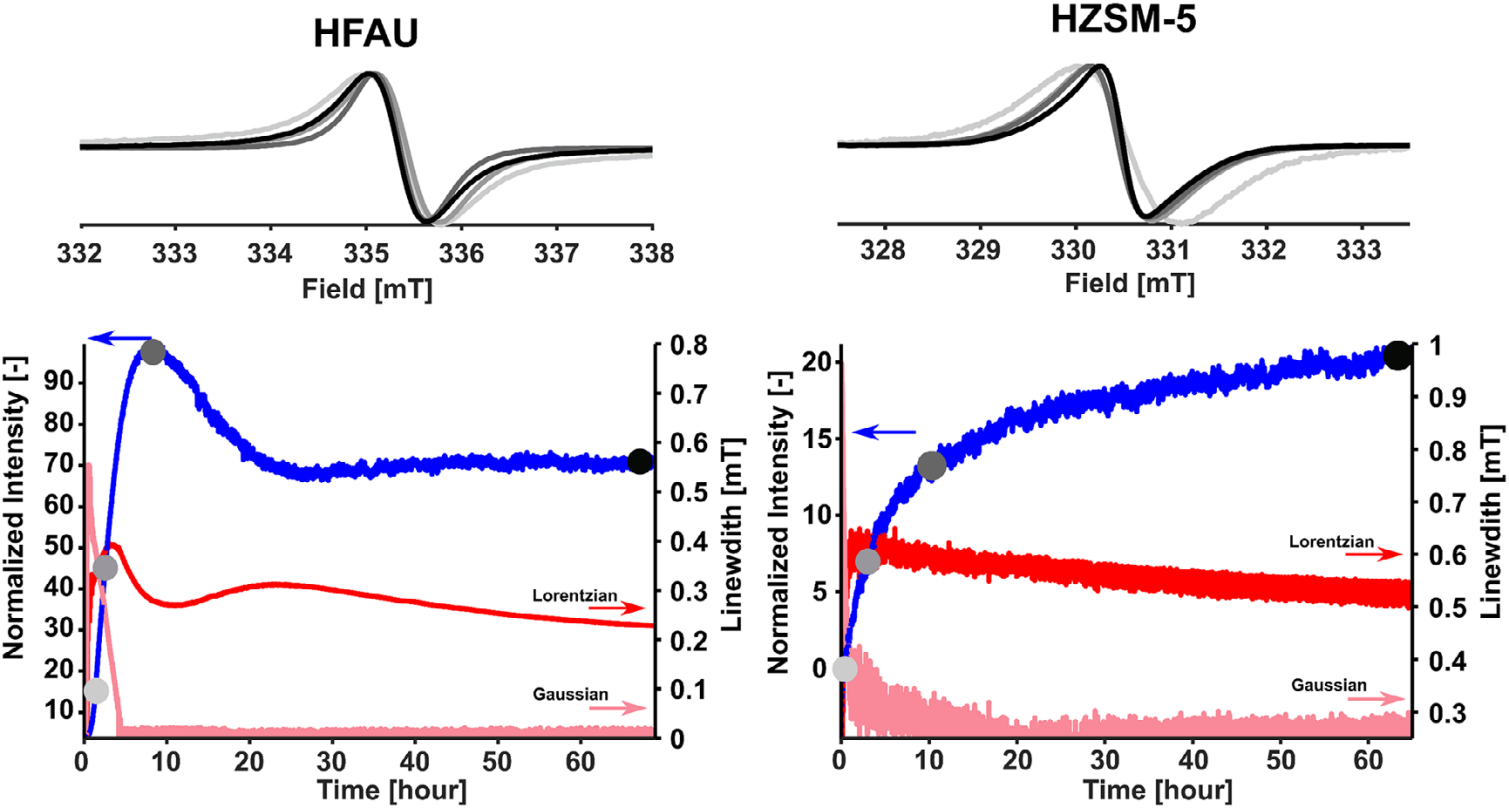
Top: Normalized ex situ EPR spectra of the respective samples HFAU (Si/Al = 2.6) and HZSM‐5 (Si/Al = 25) at certain points in time (light grey to black), as indicated by the dots in the bottom panels. Bottom: *Operando* EPR at 753 K data during the reaction for both materials. The blue trace indicates the double integral intensity, whereas the pink and red traces are the corresponding peak‐to‐peak linewidths of the spectra; the Gaussian contribution to the Voigt profile is shown in pink and the Lorentzian contribution in red.

The top panel of Figure 1 shows ex situ EPR spectra recorded from samples after a specific reaction time of phenol pyrolysis at 753 K (experimental in Scheme S1), indicated by the color gradient (additional *operando* EPR data in Figures S1 and S2). XRD patterns of the very same samples and fresh HZSM‐5 and HFAU show no indication of significant structural aging in either framework (Figure S3). All the spectra consist of a single symmetric line with a *g*‐value of 2.0075, typical for carbon‐based organic radicals [23]. The EPR signal provides insights into carbonaceous deposit formation on the zeolite catalyst, despite radicals comprising only a small fraction of the carbon atoms and the heterogeneous nature of these deposits [23, 24]. The ex situ measurements reveal a narrowing of the spectral lines between the start and endpoint of the reaction. For the HFAU (Si/Al = 2.6) samples, the spectral line shape first becomes narrower, then broadens and subsequently narrows again, contrary to the signal arising from HZSM‐5 (Si/Al = 25), which exhibits continuous spectral narrowing.

The line shape of the coke signal can generally be described by a Voigt profile with varying ratios of Lorentzian and Gaussian contributions [21]. The Gaussian component is commonly attributed to unresolved hyperfine interactions with nearby protons. A narrowing of the Gaussian linewidth thus indicates a reduction in hyperfine coupling, which suggests increased delocalization of the electron across an expanding aromatic system. The Gaussian contribution is the reason for the often‐observed linear relationship between linewidth and proton content [27]. The Lorentzian component typically reflects exchange narrowing of the linewidth due to the strong spin‐spin interaction, and its narrowing can therefore be associated with an increasing density of coke radicals.

The evolution of the EPR signal during *operando* measurements is shown in Figure 1, and the obtained traces by mass spectrometry of gases exiting the reaction system (Scheme 1) are presented in Figures S4–S6. The MS traces include phenol (C_6_H_5_OH, *m/z* 94) and the expected products acetylene (C_2_H_2_, *m/z* 26), cyclopentadiene (C_5_H_6_, *m/z* 66), benzene (C_6_H_6_, *m/z* 78), and traces of toluene (C_6_H_5_CH_3_, *m/z* 92). Line shape analysis of the EPR spectra shows that within the first 5 h, the Gaussian contribution to the linewidth decreases sharply. This decrease in the Gaussian contribution aligns with the expected condensation and growth of polyaromatic molecules, indicating the formation of initial coke on HFAU and HZSM‐5. As carbon continuously condenses, the deposits become larger, resulting in a more delocalized electronic system and a reduced hyperfine interaction. These deposits, if spatially close, result in strong local magnetic interactions that result in a Lorentzian‐type line shape, which is dominant in spectra of both catalysts after about 5 h. For HFAU, the linewidth initially decreases but begins to increase after approximately 10 h of reaction time, followed by a renewed narrowing around 25 h of time on stream. This evolution is reflected in the temporal change of the double integral (blue trace) of the coke signal, which decreases as the signal broadens and then increases again when the signal narrows. The observed correlation between a reduced double integral and an increased Lorentzian linewidth suggests that coke radicals undergo combination or disproportionation, forming diamagnetic species. This would reduce the number of radicals and weaken spin‐spin interactions, leading to a broader and less intense EPR signal [10, 16]. Since coke is believed to preferentially form in the Al‐rich pores and is reported to start in the vicinity of the product centers, it can be hypothesized that, in the large cages within the HFAU framework, multiple coke molecules can form at the same time during an initial time, but later combine due to the confinement by the cage [28, 29]. In contrast, phenol decomposition leads to the formation of less radical‐type intermediates over HZSM‐5, which results in a steady decline in Lorentzian linewidth, while the double integral is increasing rapidly until approximately 10 h, followed by a more linear increase throughout the reaction. At the end of the reaction, the final linewidth is about half as broad for HFAU as it is for HZSM‐5, which is in line with the total coke content measured by elemental analysis in HFAU, approximately two times larger than in HZSM‐5 (Figure S7). Suggesting that the linewidth can serve as an additional indicator for estimating the relative coke content. As HZSM‐5 and HFAU exhibit comparable Brønsted acid site densities (Table S1), the markedly higher carbon deposition observed on HFAU can be primarily attributed to differences in pore size and topology. The larger pore openings and three‐dimensional supercage framework of HFAU substantially enhance molecular diffusion, which improves the accessibility of molecules on active sites. This makes the reactions more frequent and accelerates the formation and accumulation of coke species. In contrast, the more restrictive pore system of HZSM‐5 limits the occurrence of reactions, thereby suppressing fast coke accumulation. While for the HZSM‐5, the coke formation and the number of observed radicals increase with the amount of coke, the observed EPR signal intensity for HFAU is not strictly correlated to the total amount of coke. (Figure 1 and Figure S7) This might lead to an underestimation of the size of the representative carbon deposits in the case of HFAU. However, we still anticipate that the detected radicals in both samples reflect topology‐dependent effects [23].

**FIGURE 2:**
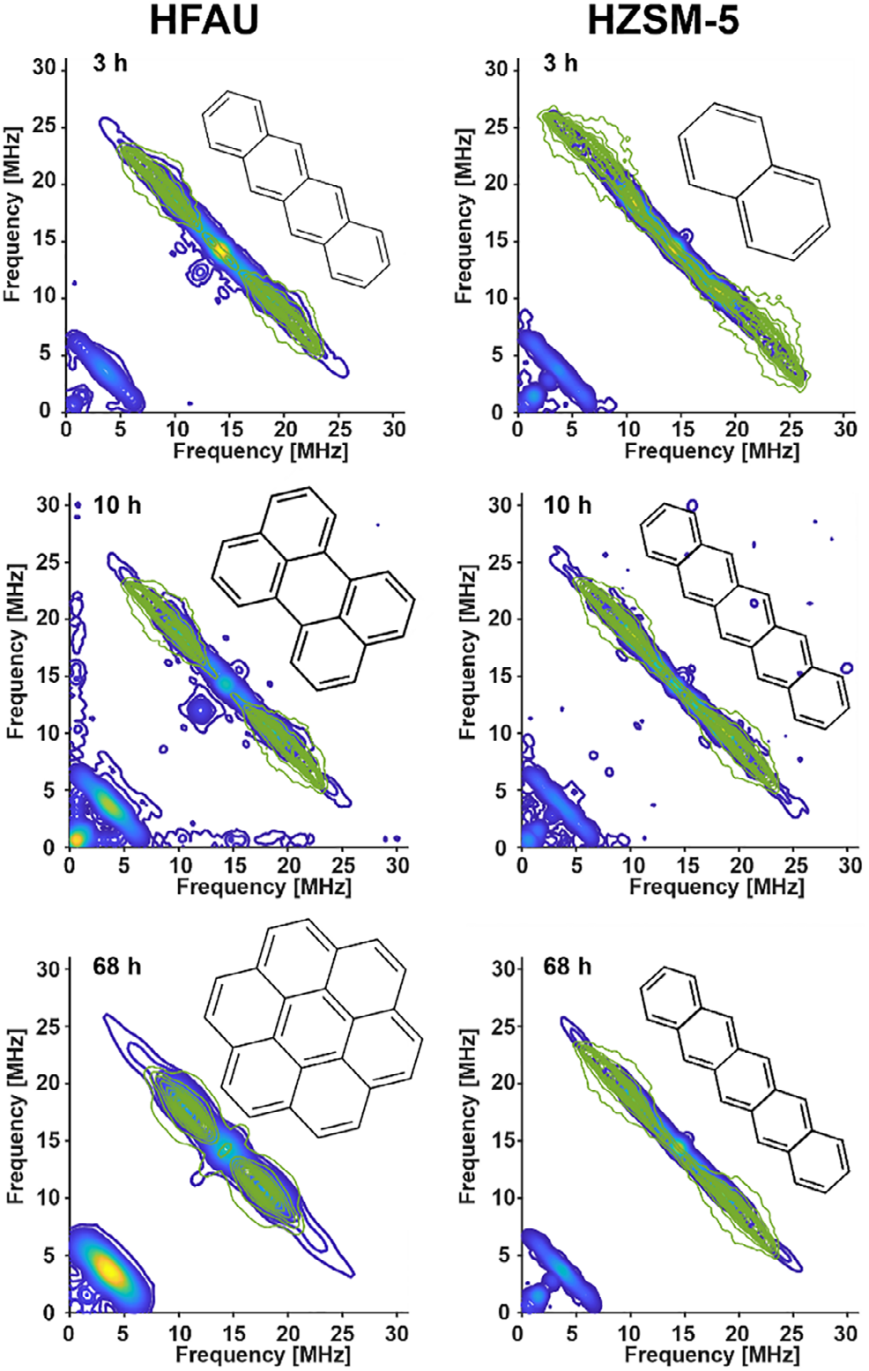
The weak interaction quadrant of the 2D HYSCORE spectra of HFAU (left) and HZSM‐5 (right) from the top to the bottom after approximately 3, 10, and 68 h reaction time. Note that the signal originated from the sample recovered after 1 h of phenol catalytic pyrolysis time was not strong enough for pulsed EPR experiments. The green lines indicate the DFT‐simulated ^1^H hyperfine couplings of different cationic molecular structures, as detailed in the insets. HFAU spectra suggest linear acenes such as tetracene at early stages evolving into non‐linear species such as perylene and coronene (C_20_H_12_ and C_24_H_12_ in the middle‐ and bottom‐left panels, respectively), whereas HZSM‐5 spectra show the initial formation of naphthalene (C_10_H_8_ in the top‐right panel) growing linearly to pentacene (C_22_H_14_ in the middle‐ and bottom‐right panels).

The samples were further characterized using hyperfine sub‐level correlation (HYSCORE) spectroscopy to elucidate the structural nature of coke deposits (Figure 2) [30]. Comparing experimental proton hyperfine patterns with DFT‐calculated patterns has proven crucial for understanding the coke structure [21, 24]. Due to the simultaneous detection of heterogeneous coke molecule distributions, the observed hyperfine patterns reflect predominantly the most abundant species, allowing for the determination of a representative coke structure.

DFT calculations were performed for 22 potential coke radicals formed during phenol catalytic pyrolysis (Table S4), and their calculated hyperfine patterns were qualitatively compared with experimental ones. We evaluated the distribution of hyperfine couplings and the relative intensity of strong and weak couplings (Figures S8–S29). Importantly, the increasingly resolved small hyperfine couplings are a fingerprint for the increase of the coke molecules (Figure S30).

The most consistent matches between theoretical and experimental patterns are presented in Figure 2. Coke species in zeolites are commonly described as cations, as the acidic zeolite environment stabilizes carbocations that subsequently evolve into trapped polyaromatic coke within the pore structure. Therefore, the DFT calculations are performed by assuming radical cations, consistent with prior literature [21, 31, 32]. Note that the coke molecules identified by HYSCORE do not account for the black color of the sample after the reaction, and are believed to be actively growing over time, acting as a “precursor” to ‘hard’ coke molecules, as graphitic coke deposits will only contribute to the matrix peaks [24]. Due to the complex coexistence of distinct radical species, the identified molecules are representative examples of the existing precursors.

For the HFAU catalyst, HYSCORE in combination with DFT suggests that the coke precursor after 3 h reaction time mostly consists of linear acenes such as tetracene. This is indicated by the pronounced ^1^H resonances with larger couplings as well as the well‐resolved small couplings. It should be noted that tetracene is possibly formed by naphthalene via the HACA mechanism or the dicyclopentadiene isomerization [33, 34, 35]. After 10 h, the proton couplings become less strong, which can be associated with a build‐up of two‐dimensional (2D) coke precursor such as perylene (C_20_H_12_), which leads to a weakening of the proton coupling due to the larger delocalization of the unpaired electron. This structure evolution can be attributed to the cage structure of HFAU, which facilitates the transformation of linear species into 2D polycyclic structures. After 68 h, the most pronounced ^1^H couplings decrease significantly, as perylene undergoes further structural growth to form larger coke precursors such as coronene (C_24_H_12_). Further evidence for the formation of hard coke is provided by the increase in intensity of the ^13^C matrix peak in the HYSCORE spectra. Even if a quantitative assessment due to the overlapping ^27^Al coupling is not possible, an increasing ratio of the ^13^C/^1^H matrix peak has been associated with the formation of hard black coke [24].

In the case of HZSM‐5, the HYSCORE spectra indicate the initial formation of naphthalene, which, constrained by the zeolite's straight‐channel structure, grows linearly to pentacene (C_24_H_14_). Nonetheless, naphthalene remains a plausible assignment for the HYSCORE spectra even after 68 h (Figure S9), consistent with the high abundance of this species we observed in previous gas‐phase catalytic pyrolysis measurements using *i*PEPICO [12]. However, it should be noted that increasingly resolved small couplings in the HYSCORE data indicate a growth of the molecules for both HZSM‐5 and HFAU (Figure S30).

These observations of coke formation in HFAU and HZSM‐5 are further supported by elemental analysis. The fraction of carbon to hydrogen atoms increases over reaction time for both zeolites (Figure S7), indicating larger amounts of coke deposits. HFAU exhibits a higher total amount of coke than HZSM‐5, consistent with the results of the EPR double integral analysis (Figure 1), suggesting that high accessibility in HFAU accelerates coke accumulation. This might originate from the restricted diffusion imposed by the narrow channels of HZSM‐5, which promotes secondary reactions, resulting in the simultaneous formation of secondary products (such as acetylene and toluene) [12, 14]. In contrast, the larger pore channels of HFAU suppress secondary transformations and favor condensation. As hydrocarbon pool species accumulate within HFAU, secondary products such as acetylene and toluene might gradually form [12, 14].

## 3 Conclusions

The here developed approach, based on *operando* EPR spectroscopy with pulsed hyperfine techniques (HYSCORE) and DFT calculations, represents a suitable methodology for unraveling coke formation and understanding key differences in the formed carbon deposits in different zeolite topologies. For HFAU, *operando* EPR data indicate that radical coke continues to grow during the catalytic pyrolysis process, with some coke molecules undergoing recombination, leading to a decrease in EPR intensity. HYSCORE spectra show that at long reaction times, bulkier coke species such as perylene and coronene dominate the coke speciation. In contrast, less coke is formed in HZSM‐5 as indicated by *operando* EPR and elemental analysis, and the EPR double integral exhibits a continuous growth. HYSCORE measurements reveal that coke precursors remain mostly linear while growing structurally, as observed from naphthalene to pentacene. The observed differences in the coke precursor in both zeolites can be correlated with the distinct topology of the respective zeolite and the confinement of the pores. While the unidirectional pore structure of HZSM‐5 constrains the species to be mostly linear, the supercages in HFAU allow for the formation of larger coke deposits. Elucidating such details is a step forward in providing valuable insights into deactivation mechanisms on HFAU and HZSM‐5 catalysts during the catalytic pyrolysis of phenol, with the potential to be extended to other processes. This knowledge can also help inform catalyst regeneration strategies and guide catalyst and process design to mitigate carbon deposition, ultimately contributing to improved catalyst longevity and process performance.

## Conflicts of Interest

The authors declare no conflicts of interest.

## Supporting information




**Supporting File 1**: Information on sample preparation and physicochemical characterization, experimental procedures, *operando* EPR spectroscopy and catalyst characterization, MS data, DFT, HYSCORE simulation, additional references [1–11], and author contributions is presented within the Supporting Information.

## Data Availability

The data that support the findings of this study are available from the corresponding author upon reasonable request.
