# Supplementary material for: Topology‐Dependent Coke Formation in the Catalytic Pyrolysis of Phenol Over HFAU and HZSM‐5 Zeolites
Source: Angew Chem Int Ed Engl. 2026 Apr 9;65(20):e23882. doi: 10.1002/anie.202523882 (PMC13159425; doi:10.1002/anie.202523882)
Supplement: Supplementary file 1 — Supporting File 1: Information on sample preparation and physicochemical characterization, experimental procedures, operando EPR spectroscopy and catalyst characterization, MS data, DFT, HYSCORE simulation, additional references [1–11], and author contributions is presented within the Supporting Information. [file ANIE-65-e23882-s001.docx]

**Supporting Information**

**Topology-Dependent Coke Formation in the Catalytic Pyrolysis of Phenol over HFAU and HZSM-5 Zeolites**

Jörg W. A. Fischer^[a],[b]+^, Allen Puente-Urbina^[c],[d],+,*^, Zeyou Pan^[c],[e],+^, Mikhail Agrachev^[a]^, Patrick Hemberger^[e]^, Gunnar Jeschke^[a],*^, Jeroen A. van Bokhoven^[b],[f]*^

[a] Dr. J. W. A. Fischer, Dr. M. Agrachev, Prof. Dr. G. Jeschke
Institute for Molecular Physical Science
ETH Zurich
Vladimir-Prelog-Weg 1-5/10, 8093 Zurich (Switzerland)
E-mail: [gunnar.jeschke@phys.chem.ethz.ch](mailto:gunnar.jeschke@phys.chem.ethz.ch)

[b] Present address: Dr. J. W. A. Fischer
Institute for Catalysis
Hokkaido University
N-21, W-10, 001-0021 Sapporo (Japan)

[c] Dr. A. Puente-Urbina, Dr. Z. Pan, Prof. Dr. J. A. van Bokhoven
Institute for Chemical and Bioengineering
ETH Zurich
Vladimir-Prelog-Weg 1-5/10, 8093 Zurich (Switzerland)
E-mail: [jeroen.vanbokhoven@chem.ethz.ch](mailto:jeroen.vanbokhoven@chem.ethz.ch)

[d] Present address: Dr. A. Puente-Urbina
School of Chemistry
Costa Rica Institute of Technology
P.O. Box 159-7050, 30101 Cartago (Costa Rica)

E-mail: [apuente@itcr.ac.cr](mailto:apuente@itcr.ac.cr)

[e] Dr. Z. Pan, Dr. P. Hemberger
Laboratory for Synchrotron Radiation and Femtochemistry
Paul Scherrer Institute
Forschungsstrasse 111, 5232 Villigen (Switzerland)

[f] Prof. Dr. J. A. van Bokhoven
Center for Energy and Environmental Sciences
Paul Scherrer Institute
Forschungsstrasse 111, 5232 Villigen (Switzerland)

[+] These authors contributed equally to this work.

**Abstract:** Catalytic pyrolysis of lignin, the most abundant natural aromatic polymer, offers a route to obtain value-added products with a low carbon footprint. In such a process, the lignin structure undergoes decomposition through an intricate network of reaction routes. Despite the use of model compounds to gain insights into the decomposition pathways, the formation mechanism of coke and its role in critically affecting catalyst performance remain poorly understood. Herein, we use *operando* electron paramagnetic resonance (EPR) spectroscopy together with ex situ pulsed EPR experiments and density functional theory (DFT) calculations to understand coke formation in catalytic pyrolysis of phenol over HFAU and HZSM-5 zeolites. Our results pinpoint that coke formation is heavily influenced by zeolite topology. The large cages in HFAU facilitate the initial formation of linear configurations that grow to extended structures, whereas the narrower channels in HZSM-5 promote the formation of more linear structures. These results provide comprehensive mechanistic insights into coke formation and growth that are relevant for the development of lignin valorization strategies and for the general phenomenon of coke formation in zeolites and beyond.**Table of Contents**

Sample Preparation and Physicochemical Characterization...…………………………….……..……….S3

Experimental Procedures………………………………………………………………………….................S3

*Operando* EPR Spectroscopy and Catalyst Characterization…………………………………….…….…S5

MS Data…………………………………………………………………..………...………………………..…S6

DFT………………………………………………………………...………………………..............................S7

HYSCORE Simulation…….…………………………………...………………………..............................S12

References…………………………………………………………………………...………………………..S35

Author Contributions………………………………………………………………...………………………..S36

**Sample Preparation and Physicochemical Characterization**

Zeolite HZSM-5 (25) and HFAU (2.6), where (..) represents the Si/Al ratio, were purchased from Zeolyst International. The zeolite was pressed, crushed, and sieved. Before the experiments, all zeolites were calcined at 823 K with a heating rate of 2 K min^−1^ in static air and then maintained at the final temperature for 6 h.

**Table S1.** Composition of the prepared zeolite materials.

| Sample name  and composition | Average poor  diameter/ Å | V_micro_ (cm^3^/g) | BAS (mmol/g) | LAS (mmol/g) | Ref. |
| --- | --- | --- | --- | --- | --- |
| HZSM-5 (Si/Al=25) | 5.4 – 5.6 | 0.15 | 0.33 | 0.064 | ^1^ |
| HFAU (Si/Al=2.6) | 7.4 | 0.27 | 0.43 | 0.24 | ^2^ |

**Experimental Procedures**

***Operando Electron Paramagnetic Resonance (EPR) Spectroscopy***


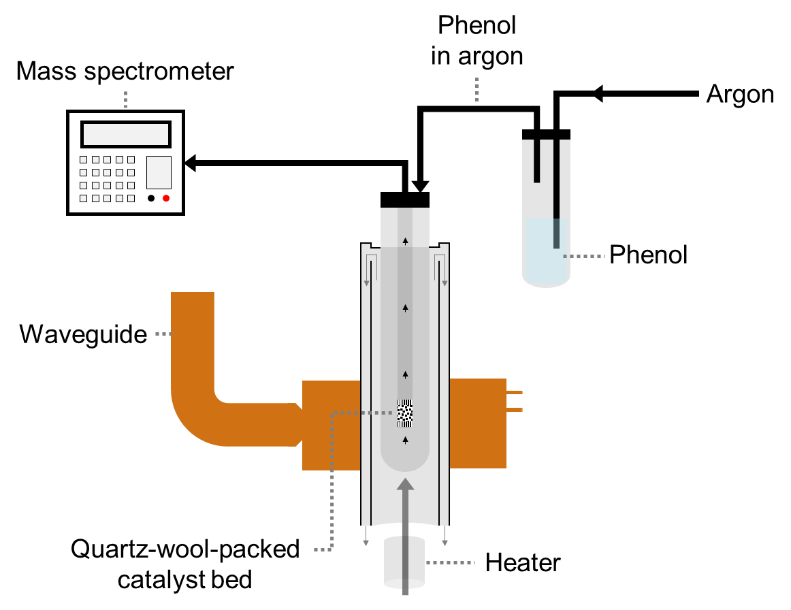
*Operando* continuous-wave (cw) EPR spectroscopy experiments were performed using a homebuilt water-cooled high-temperature resonator, which was installed into a continuous-wave (cw) EPR spectrometer (Bruker EMX) operating at X-band frequencies (~9.265 GHz). The resonator is heated by a flow of hot N_2_ using a home-built temperature controller. The flow-through cell consists of two aligned quartz tubes. Inside the EPR quartz reactor (Wilmad; 4 mm OD), a thinner inner capillary (Qsil: 2.0-2.4 mm OD) is inserted, and this assembly is connected to the gas supply system.^3^ The catalyst bed (ca. 10 mg) was fixed between two quartz wool plugs in the inner capillary to ensure maximal contact with the reaction gas mixture. The experimental setup consisted of mass flow controllers (Bronkhorst) to control the gas flows (Scheme S1). A total flow rate of 10 mL min^-1^ was used for the EPR experiments. Note that, due to the intrinsic limitations of the *operando* EPR setup used in this study, with its connecting tubes remaining at room temperature, the MS traces cannot be interpreted quantitatively.

**Scheme S1**. Experimental setup for the study of phenol pyrolysis using *operando* EPR.

The room-temperature CW EPR experiments were carried out in a 3 mm OD quartz tube (Wilmad) on a continuous-wave (CW) EPR spectrometer (Bruker EMX) operating at X-band frequencies, equipped with a Super-High-Q resonator (Bruker Biospin) at ~9.5 GHz.

**Table S2.** Measurement parameters for the *operando* X-band cw EPR measurements.

| Experiment | Sweep  width [mT] | Modulation frequency [kHz] | Modulation amplitude [mT] | Sweep  time [s] | Conversion  time [ms] | Time  constant [ms] | Power  attenuation [dB] |
| --- | --- | --- | --- | --- | --- | --- | --- |
| HFAU | 10 | 100 | 0.1 | 30 | 50 | 20.4 | 26 |
| HZSM5 | 40 | 100 | 0.1 | 60 | 40 | 40.9 | 20 |

**Table S3.** Measurement parameters for the room temperature X-band cw EPR measurements.

| Experiment | Sweep  width [mT] | Modulation frequency [kHz] | Modulation amplitude [mT] | Repetition  time [s] | Time  constant [ms] | Power  attenuation [dB] |
| --- | --- | --- | --- | --- | --- | --- |
| Room temperature | 40 | 100 | 0.2 | 80 | 40 | 30 |

To gain insight into the density of coke deposits, these spectra were least-squares fitted to extract their Gaussian and Lorentzian contributions. While dense deposits result in local magnetic interactions producing a Lorentzian-type EPR signal, virtually isolated paramagnetic species generate an EPR spectrum with a Gaussian lineshape. Previous work suggested that this narrowing is related to an increased electron spin density delocalization, which is linked to the growth of polyaromatic molecules.^4–6^ Accordingly, the cw EPR spectra were linearly baseline-corrected and subsequently simulated via EasySpin routines as a Voigt profile by varying the weight of the Lorentzian and Gaussian contributions.^7^ To avoid the fit getting stuck in a local minimum, the computation consists of 1 minute of Monte Carlo simulations and subsequent application of a Simplex algorithm to find the global minimum.

The intensity of the cw EPR spectrum is proportionally related to the first derivative of the magnetic susceptibility of the sample at a certain temperature and external magnetic field strength. Accordingly, the double integral of the EPR spectrum is a direct measure of the total number of spins in the sample. The standard procedure to determine the concentration is double integration of the spectrum.^8^ Here, the relative increase of the coke intensity during phenol decomposition was determined by double integration of the difference spectra at each point in time throughout the reaction. The difference spectra were computed by subtracting the spectrum of the pure zeolite before phenol dosing at 753 K in air.

***Ex situ* Electron Paramagnetic Resonance (EPR) Spectroscopy and DFT calculations**

2D HYSCORE spectroscopy was performed at 50 K on a Bruker EleXsys E580 spectrometer using a standard split-ring resonator (MS3) and an Oxford helium (CF 935P) cryostat at a frequency of approximately 9.6 GHz. The microwave pulses were amplified using a 1 kW pulsed traveling wave tube (TWT) amplifier. The measurements were conducted by applying a π/2-τ-π/2-t_1_-π-t_2_-π/2-τ echo pulse sequence with π/2 pulse length of 24 ns and an inversion π pulse length of 16 ns with time delays, τ, of 100, 124, and 224 ns to avoid blind-spot artifacts. The 2D HYSCORE time-domain data were recorded by measuring the echo amplitude as a function of dimensions t_1_ and t_2_. The pulse sequence was repeated in an 8-step phase cycling procedure to avoid undesired echoes.^9^ The obtained spectra were then processed with the software package HYSCOREAN.^10^ Note that the spectrum for HFAU after 68 h time on stream has been recorded with half the points as the other data, resulting in a lower resolution in the frequency domain. The simulations of the hyperfine interactions of different molecular structures were performed applying Kohn-Sham density functional theory and using a B3LYP functional with a spin-unrestricted shell and a 6-31G++(d,p) basis set in the Gaussian software.^11^ Prior to the DFT calculations, all molecular structures were optimized using the semi-empirical Gaussian method employing PM3 in analogy to the approach used by Cesarini et a..^6^ All the 2D HYSCORE spectra were simulated using only non-equivalent nuclei to rationalize computational cost with EasySpin routines, with the hyperfine couplings from the DFT calculations.^7^

***Operando* EPR Spectroscopy and Catalyst Characterization**


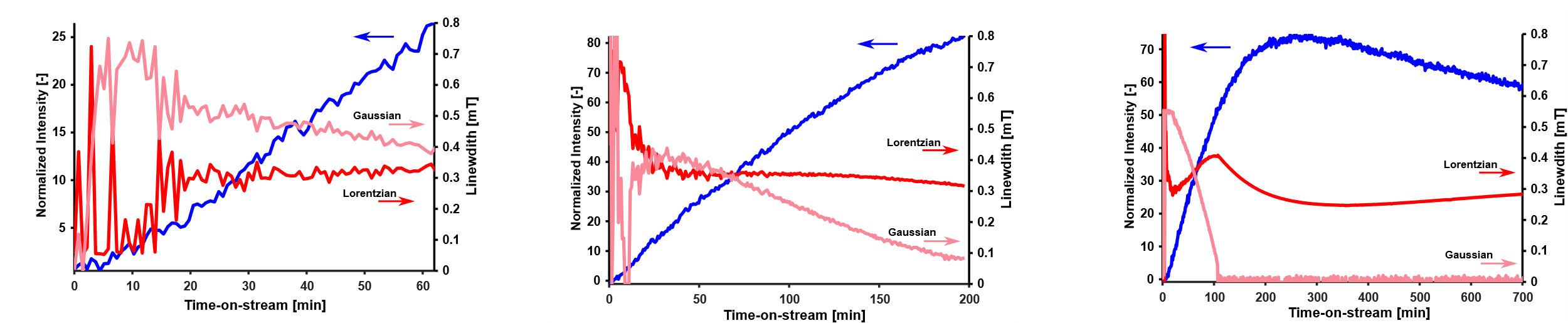


**Figure S1** *Operando* EPR data of coke formation in HFAU during the reaction. In blue the double integral intensity and in red the two components of the Voigt profile.


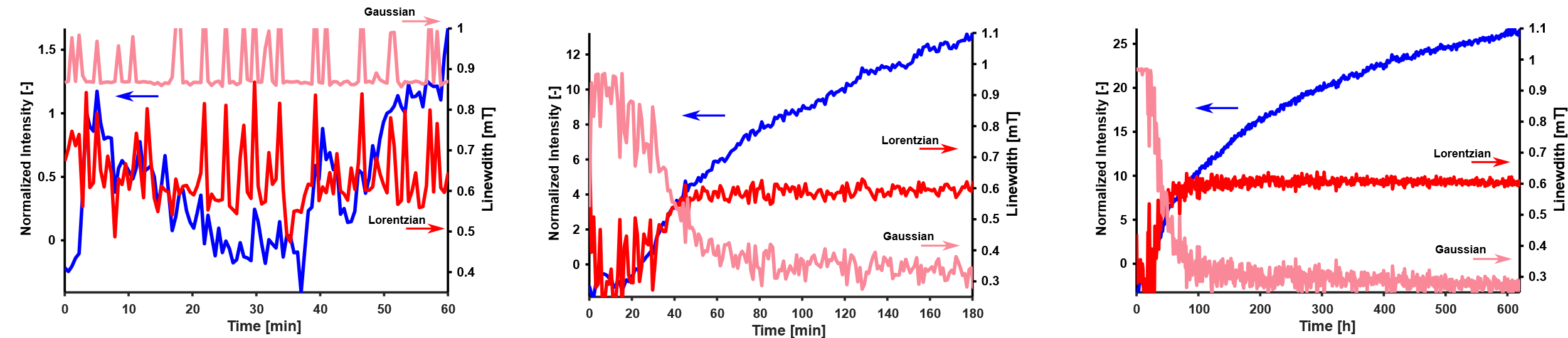


**Figure S2** *Operando* EPR data of coke formation in HZSM-5 during the reaction. In blue the double integral intensity and in red the two components of the Voigt profile.


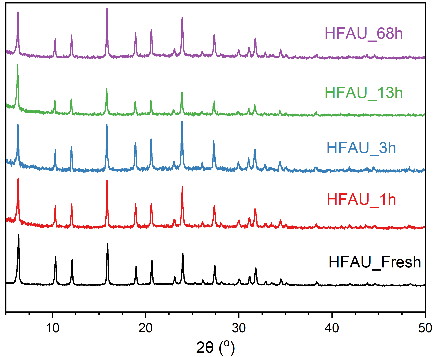

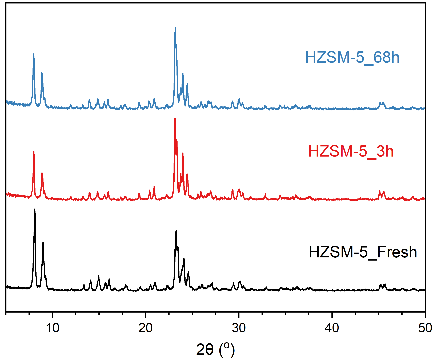
Figure S3. PXRD patterns of HFAU (left) and HZSM-5 (right)

**MS Data**

It should be noted that the connecting tubes remained at room temperature during the *operando* EPR measurements, which prevented the MS traces from being assessed quantitatively, as condensation of reactants and products could occur during the reaction. This might also be the reason for the unexpected increase of phenol at around 30 h in both reactions, which might stem from slow saturation of the atmosphere in the tubes. As the other observed fragments remain more or less stable, a direct link to the deactivation of the catalyst cannot be drawn. It should be further noted that the m/z = 66 and m/z = 26 fragments are also a fragmentation signal of pure phenol from the ionization in the MS itself. However, as they do not increase as a result of the increase in the phenol signal around 30 h (Fig. S7), they cannot be purely attributed to phenol. At the same time, m/z = 78 and m/z = 92 are not ionization fragments of phenol, and indicate reactivity.

**
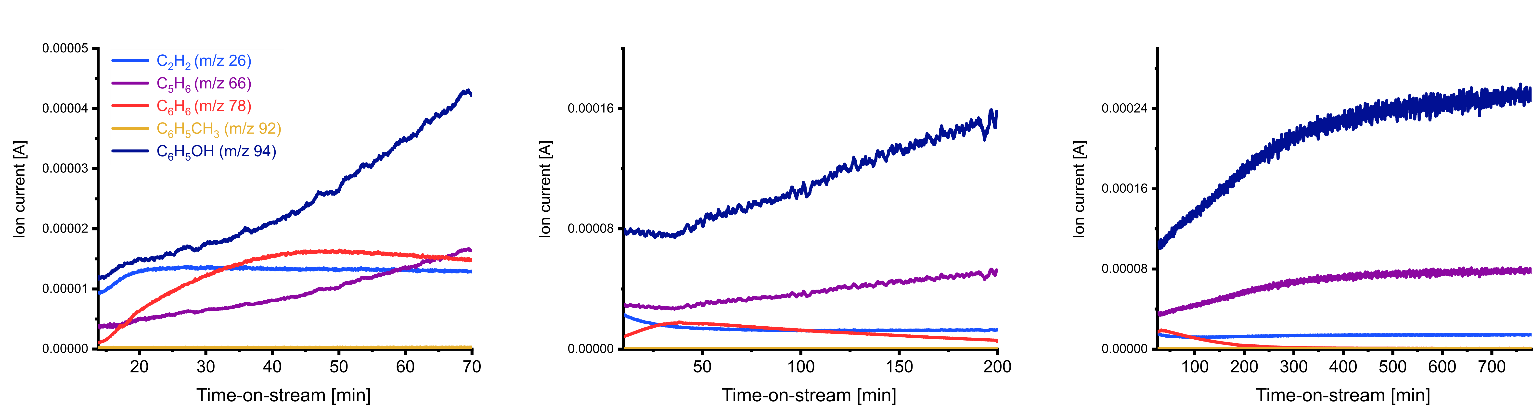
**

Figure S4. MS traces of the phenol decomposition over HFAU for different times on stream according to Scheme 1.
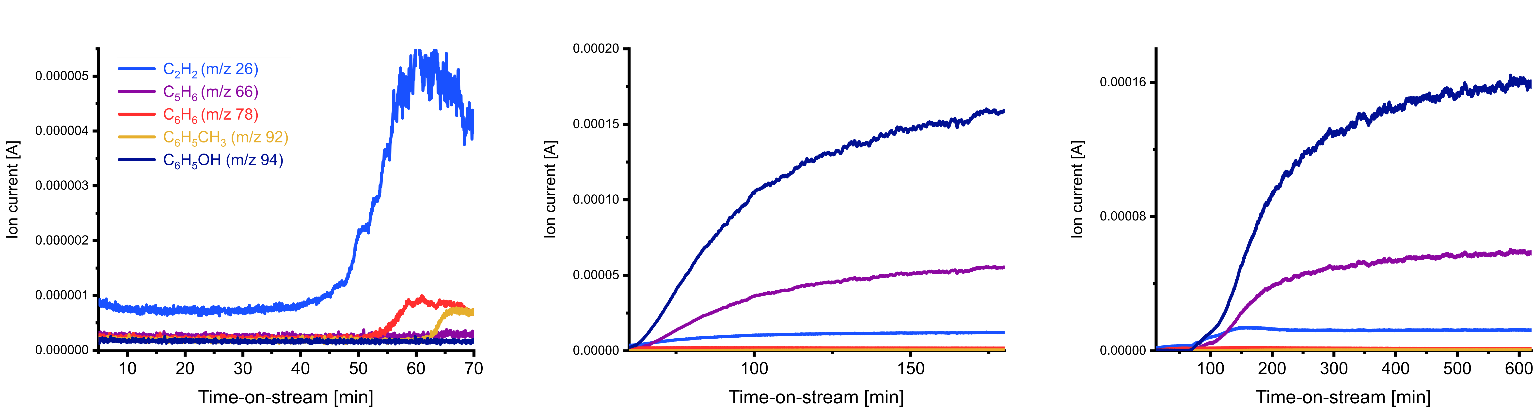


Figure S5. MS traces of the phenol decomposition over HZSM-5 for the different times on streams according to Scheme 1.


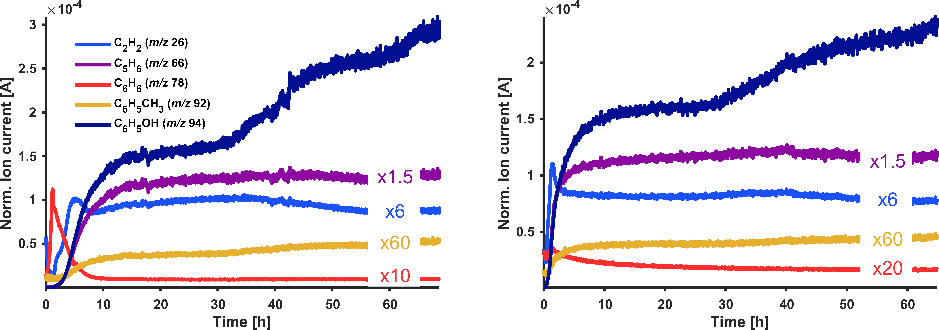
Figure S6. MS traces of the phenol decomposition over HZSM-5 and HFAU for 68 h according to Scheme 1.

**Elemental analysis**

Figure S7. Elemental analysis data of HFAU (left) and HZSM-5 (right)


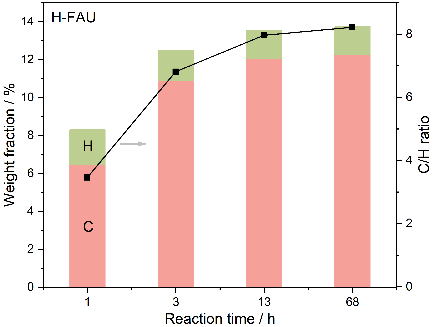

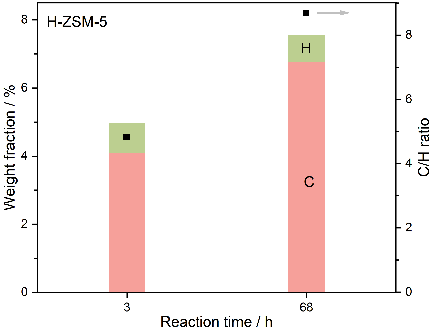


**DFT**

**Table S4.** Hyperfine interactions of the cationic molecular structures extracted from DFT.

| Structure | ^Name^ | ^1^H_x_ | Isotropic Fermi contact coupling  A (MHz) | Anisotropic spin dipole coupling  Tx (MHz) Ty (MHz) Tz (MHz) | | |
| --- | --- | --- | --- | --- | --- | --- |
| 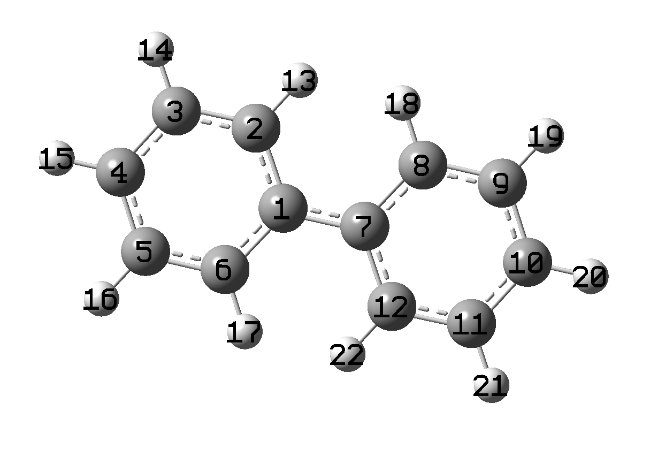 | Biphenyl | H_13_ H_17_ H_18_ H_22_  H_14_ H_16_ H_19_ H_21_  H_15_ H_20_ | -8.56850  2.26643  -17.59130 | -3.903  -1.976  -10.582 | -2.443  -0.297  -1.437 | 6.346  2.273  12.018 |
| 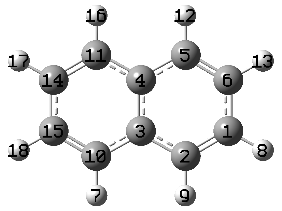 | Naphthalene | H_7_ H_9_ H_12_ H_16_  H_8_ H_13_ H_17_ H_18_ | -15.55670  -4.36562 | -8.347  -3.653 | -2.505  -2.142 | 10.852  5.795 |
| 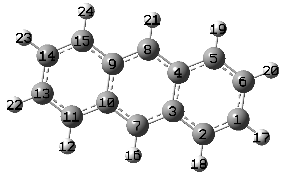 | Anthracene | H_12_ H_18_ H_19_ H_24_  H_16_ H_21_  H_17_ H_20_ H_22_ H_23_ | -8.87411  -17.63704  -3.12260 | -4.000  -9.476  -2.569 | -1.861  -2.166  -1.342 | 5.861  11.642  3.911 |
| 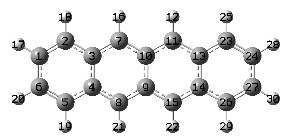 | Tetracene | H_12_ H_16_ H_21_ H_22_  H_17_ H_20_ H_28_ H_30_  H_18_ H_19_ H_25_ H_29_ | -13.85586  -2.34657  -5.21382 | -7.074  -1.898  -1.935 | -1.871  -0.925  -1.409 | 8.945  2.824  3.344 |
| 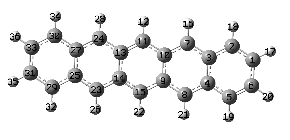 | Pentacene | H_12_ H_22_  H_16_ H_21_  H_17_ H_20_  H_18_ H_19_  H_26_ H_28_  H_32_ H_34_  H_35_ H_36_ | -12.36885  -9.46115  -1.5664  -2.94016  -2.94016  -4.14002  -1.90129 | -6.421  -4.872  -1.365  -0.969  -6.035  -1.529  -1.630 | -1.629  -1.314  -0.594  -0.908  -1.460  -1.118  -0.699 | 8.049  6.186  1.959  1.878  7.494  2.647  2.329 |
| 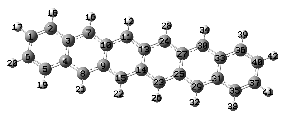 | Hexacene | H_12_ H_22_ H_26_ H_28_  H_16_ H_21_ H_32_ H_34_  H_17_ H_20_ H_41_ H_42_  H_18_ H_19_ H_38_ H_39_ | -12.48387  -8.24758  -1.32734  -2.36683 | -5.773  -3.601  -1.060  -0.759 | -1.252  -1.030  -0.451  -0.525 | 7.025  4.631  1.510  1.284 |
| 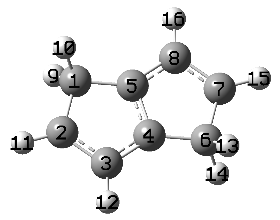 | Pentalene | H_9_ H_10_ H_13_ H_14_  H_11_ H_15_  H_12_ H_16_ H_38_ H_39_ | -1.97649  -28.49605  2.08138 | -3.771  -16.580  -2.730 | -0.096  -1.867  -1.106 | 3.868  18.447  3.836 |
| 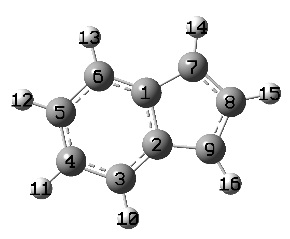 | Indene | H_10_ H_13_  H_11_ H_12_  H_14_ H_16_  H_15_ | -6.95191  -3.70287  -32.22955  8.37392  -3.568 | -2.644  -2.689  -19.258  -3.568 | -1.941  -1.215  -1.395  -0.600 | 4.585  3.904  20.654  4.168 |
| 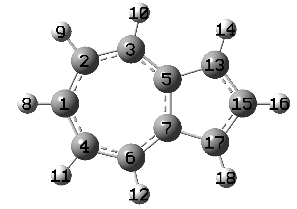 | Azulene | H_8_  H_9_ H_11_  H_10_ H_12_  H_14_ H_18_  H_16_ | 3.98582  -11.48717  1.82925  -28.98052  6.07063 | -1.550  -6.639  -2.584  -17.043  -3.197 | -0.363  -0.996  -1.080  -2.115  0.495 | 1.913  7.635  3.664  19.158  2.702 |
| 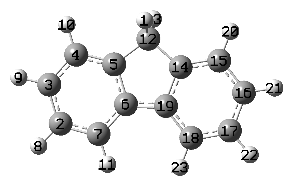 | Fluorene | H_1_ H_13_  H_8_ H_22_  H_9_ H_21_  H_10_ H_20_  H_11_ H_23_ | 2.24535  0.80332  -17.73942  3.60464  -7.19439 | -2.301  -1.947  -10.707  -2.046  -3.847 | -0.429  -0.970  -1.427  -0.133  -2.062 | 2.730  2.917  12.134  2.179  5.910 |
| 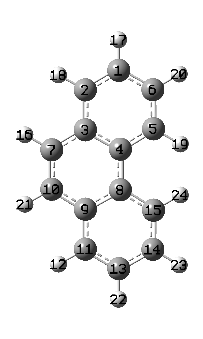 | Phenanthrene | H_12_ H_18_ H_20_ H_23_  H_16_ H_21_  H_17_ H_22_  H_19_ H_24_ | -11.26696  -12.81099  3.53290  -1.57055 | -5.770  -7.316  -1.585  -1.733 | -1.685  - -2.551  -0.218  -1.052 | 7.456  9.867  1.802  2.786 |
| 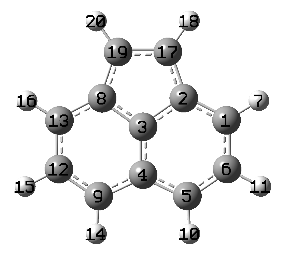 | Acenaphthylene | H_7_ H_16_  H_10_ H_14_  H_11_ H_25_  H_18_ H_20_ | --2.568  -7.785  -0.7692  -20.440 | -1.534  -4.375  -1.215  -12.484 | -1.527  -1.126  -0.508  -2.844 | 3.060  5.501  1.723  15.328 |
| 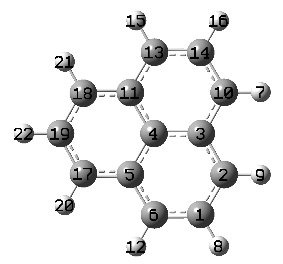 | Phenalene | H_7_ H_9_ H_12_ H_15_ H_21_ H_21_  H_8_ H_16_ H_22_ | -18.13268  6.44992 | -3.479  -0.812 | -0.417  -0.438 | 3.896  1.249 |
| 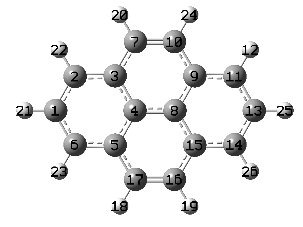 | Pyrene | H_12_ H_22_ H_23_ H_26_  H_18_ H_19_ H_20_ H_24_  H_21_ H_25_ | -15.26180  -5.68760  4.41306 | -8.648  -2.523  -1.911 | -1.369  -1.782  -0.386 | 10.017  4.306  2.297 |
| 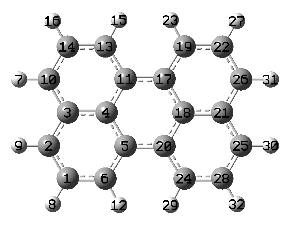 | Perylene | H_7_ H_9_ H_30_ H_31_  H_8_ H_16_ H_27_ H_32_  H_12_ H_15_ H_23_ H_29_ | -11.86619  2.07905  -8.43155 | -2.223  -0.516  -1.444 | -0.458  -0.093  -0.539 | 2.681  0.609  1.984 |
| 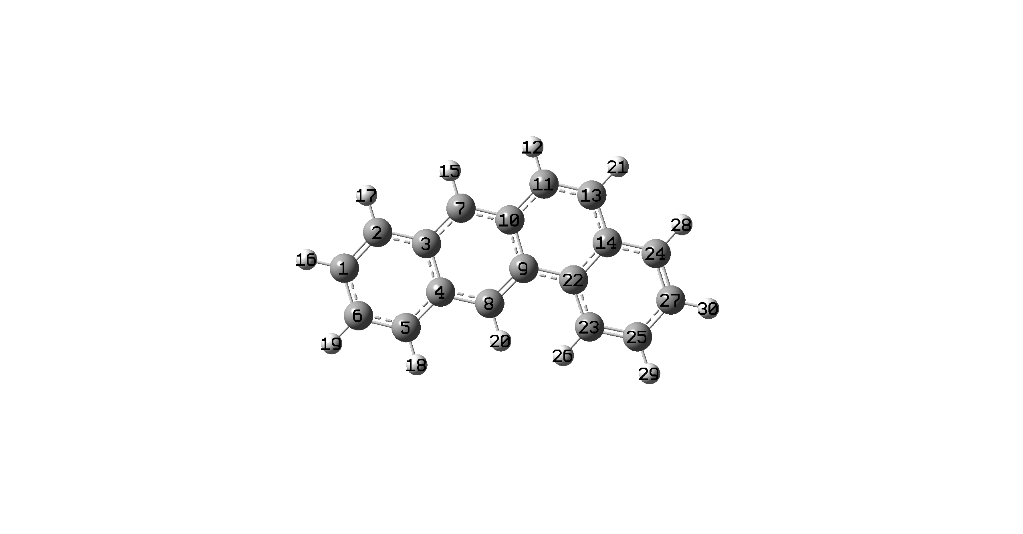 | Tetraphene | H_12_  H_15_  H_16_  H_17_  H_18_ H_19_  H_20_  H_21_  H_26_  H_28_  H_29_  H_30_ | -7.96700  -17.75560  -0.29214  -10.10891  -5.76504  -13.08283  -6.10316  2.35228  -2.26668  -5.84024  0.41970 | -3.470  -9.462  -1.374  -4.681  -2.621  -7.164  -3.951  -1.506  -1.291  -3.486  -0.773 | -2.077  -2.169  -1.146  -1.703  -1.710  -1.709  -1.536  -1.341  -0.952  -0.592  -0.368 | 5.548  11.631  2.519  6.384  4.331  8.873  5.487  2.847  2.243  4.078  1.141 |
| 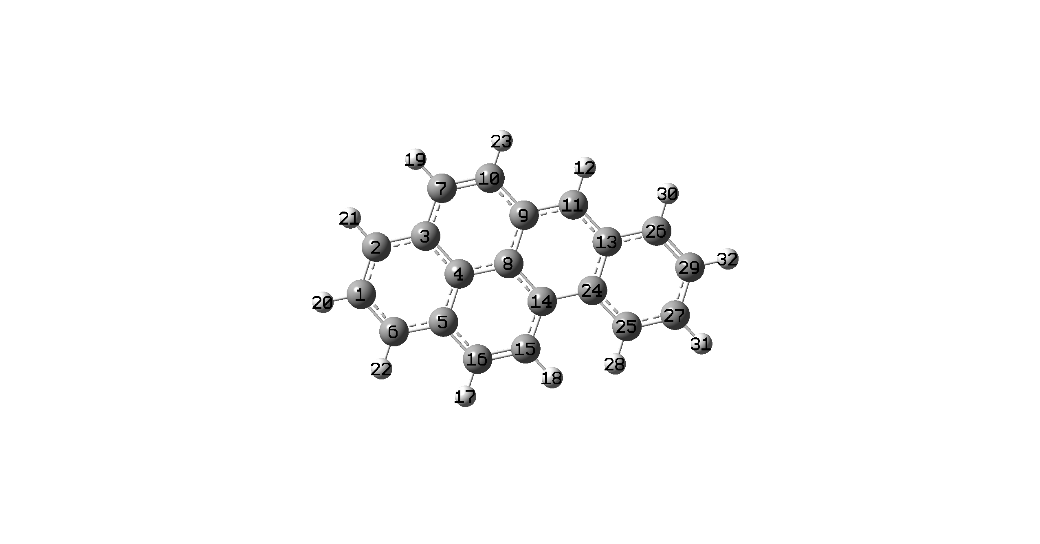 | Benzopyrene | H_12_  H_17_ H_31_  H_18_  H_19_ H_23_  H_20_  H_21_  H_22_  H_28_  H_30_  H_31_ | -18.63560  -7.64186  0.08404  -4.77450  3.10653  -10.49360  -12.88348  -0.92612  -8.78764  -7.42106 | -10.216  -3.695  -1.340  -2.343  -1.513  -5.927  -7.179  -1.272  -3.823  -4.546 | -1.864  -1.238  -0.281  -1.539  -0.270  -1.097  -1.101  -0.464  -1.407  -0.706 | 12.080  4.932  1.621  3.882  1.783 7.025  8.281  1.736  5.230  5.251 |
| 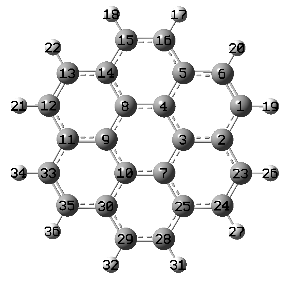 | Coronene | H_17_ H_18_ H_31_ H_32_  H_19_ H_21_ H_26_ H_34_  H_20_ H_22_ H_27_ H_36_ | -6.13418  3.29227  -9.04298 | -3.593  -1.245  -4.982 | -1.504  -0.484  -1.041 | 5.097  1.729  6.023 |
| 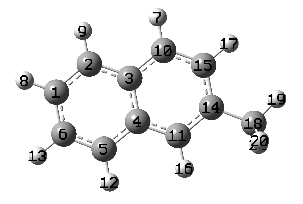 | Methylnaphthalene | H_7_  H_8_  H_9_  H_12_  H_13_  H_16_  H_17_  H_19_  H_20_  H_21_ | -15.3946  -8.28734  -11.93870  -17.43857  -0.06790  -18.02691  0.62965  -0.82201  28.40577  23.40037 | -8.463  -5.554  -6.387  -9.118  -2.076  -9.725  -1.973  -1.111  -2.327  -2.326 | -2.060  -1.798  -2.362  -2.341  -1.573  -2.808  -0.866  -1.057  -0.528  -0.523 | 10.523  7.352  8.749  11.459  3.649  12.534  2.839  2.168  2.855  2.848 |
| **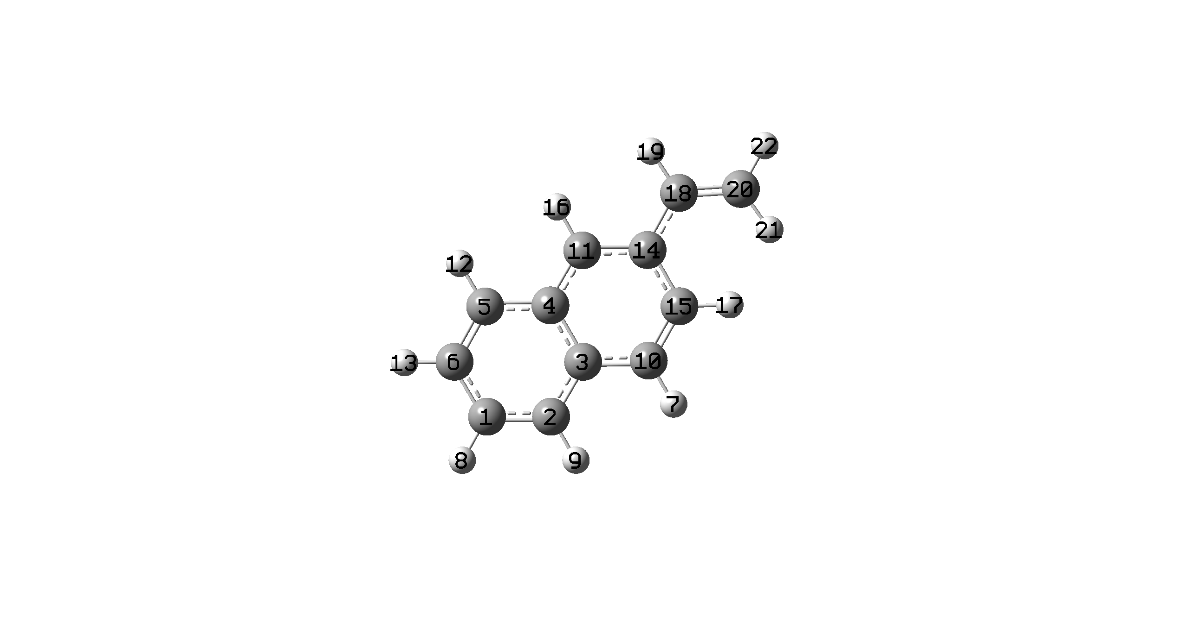** | Ethylnaphthalene | H_7_  H_8_  H_9_  H_12_  H_13_  H_16_  H_17_  H_19_  H_21_  H_22_ | -8.36905  -11.36773  -4.76578  -14.67418  3.17865  -15.43088  2.65186  -17.91970  -18.59814 | -4.694  -7.030  -2.862  -7.541  -1.806  -8.316  -2.155  -2.862  -10.008  11.125 | -1.415  -1.313  -1.891  -1.879  -0.434  -2.559  -0.955  -0.584  -1.644  -1.365 | 6.109  8.343  4.753  9.420  2.241  10.875  3.111  3.446  11.652  12.490 |
| 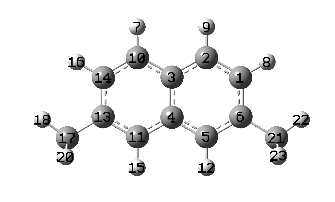 | Dimethylnaphthalene | H_7_ H_9_  H_8_ H_16_  H_12_ H_15_  H_18_ H_22_  H_19_ H_23_  H_20_ H_24_ | -13.13802  -2.82248  -19.78631  -1.05772  14.73074  13.62234 | -7.186  -2.517  -10.517  -0.982  -1.917  -1.927 | -2.075  -1.771  -2.719  -0.788  -0.596  -0.608 | 9.261  4.287  13.23  1.770  2.513  2.536 |
| 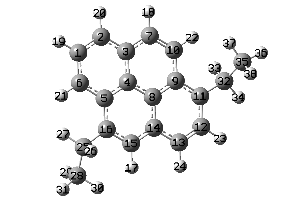 | Dieethylpyrene | H_17_  H_18_  H_19_ H_23_ H_27_  H_20_ H_21_  H_22_  H_24_  H_26_  H_30_  H_31_  H_33_  H_34_  H_36_  H_37_ H_38_ | -6.17256  -4.72494  4.01385  -13.13302  -3.84618  -14.03652  20.71286  -0.44510  0.60661  1.94150  24.91092  0.84529  3.35815  -1.25480 | -2.926  -2.038  -1.702  -7.480  -1.607  -7.928  -1.185  -1.185  -0.579  -1.777  -1.290  -1.077  -1.629 | -2.034  -1.515  -0.408  -1.140  -1.197  -1.405  -0.719  -0.596  -0.437  -0.788  -1.197  -0.639  0.110 | 4.960  3.553  2.109  8.620  2.803  9.333  1.904  1.781  1.017  2.564  2.487  1.717  1.519 |

**HYSCORE Simulation**

**
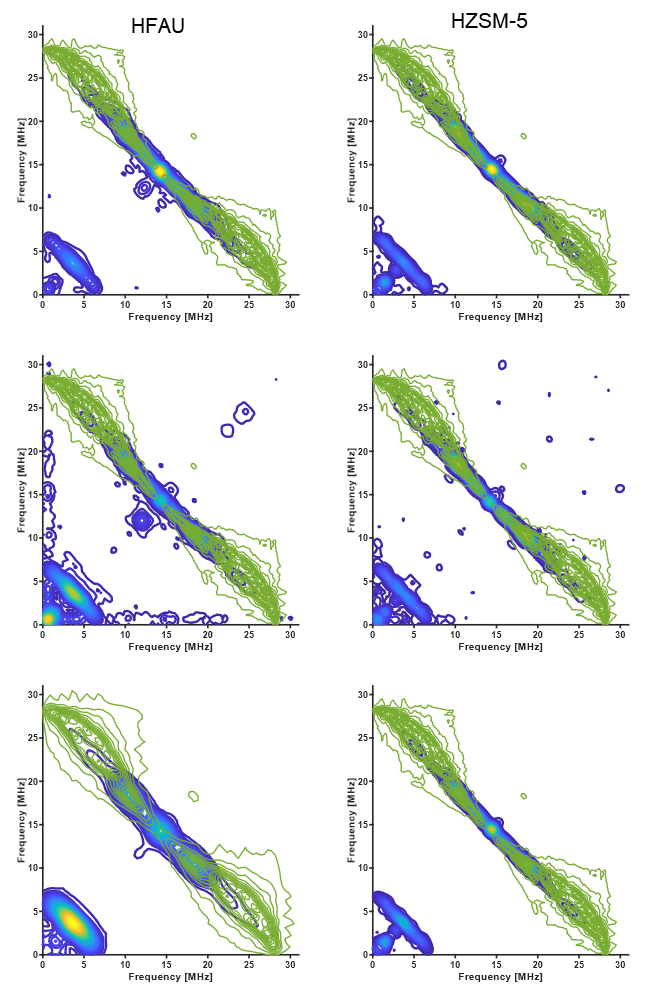
**

**Figure S8.** The weak interaction quadrant of the 2D HYSCORE spectra of HFAU (left) and HZSM-5 (right) from the top to the bottom after ~3 h, ~10 h, ~68 h reaction time. The green lines indicate the DFT simulated hyperfine couplings of Biphenyl cation radical.

**
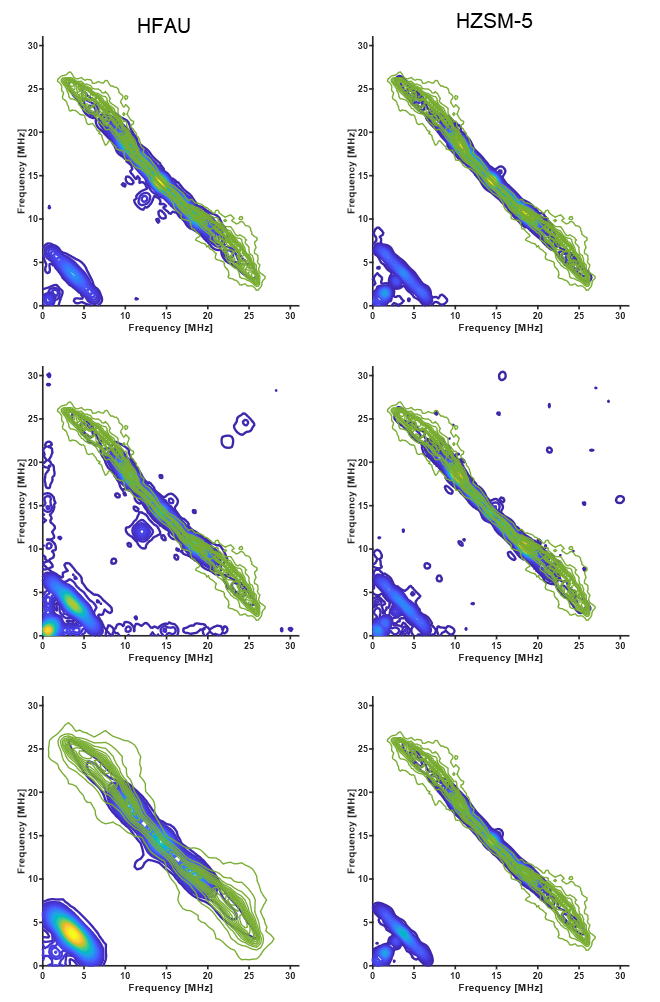
Figure S9.** The weak interaction quadrant of the 2D HYSCORE spectra of HFAU (left) and HZSM-5 (right) from the top to the bottom after ~3 h, ~10 h, ~68 h reaction time. The green lines indicate the DFT simulated hyperfine couplings of Naphthalene cation radical.


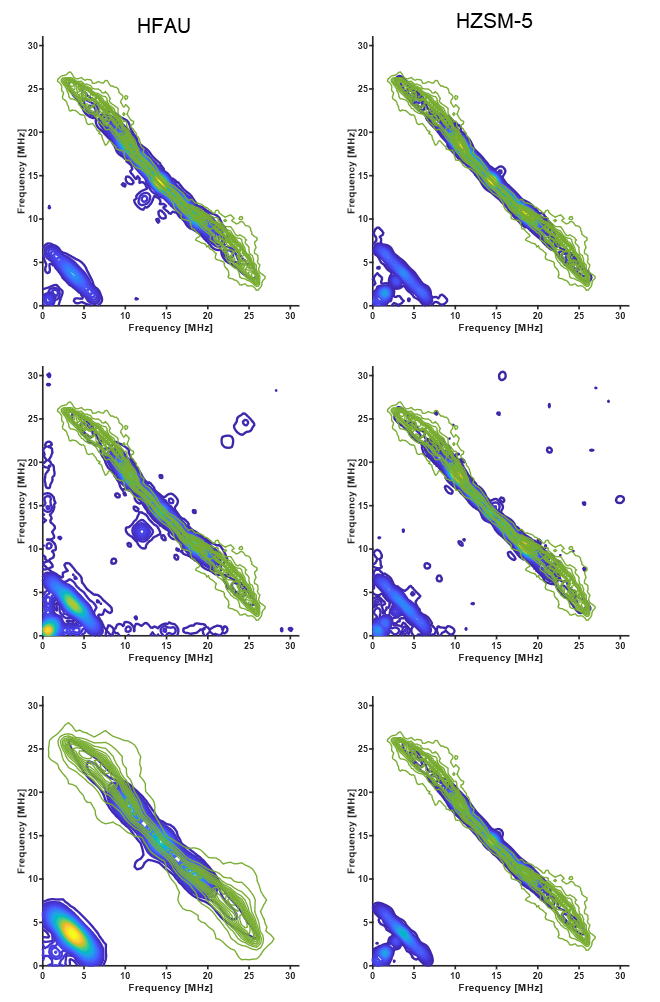
**Figure S10.** The weak interaction quadrant of the 2D HYSCORE spectra of HFAU (left) and HZSM-5 (right) from the top to the bottom after ~3 h, ~10 h, ~68 h reaction time. The green lines indicate the DFT simulated hyperfine couplings of Anthracene cation radical.


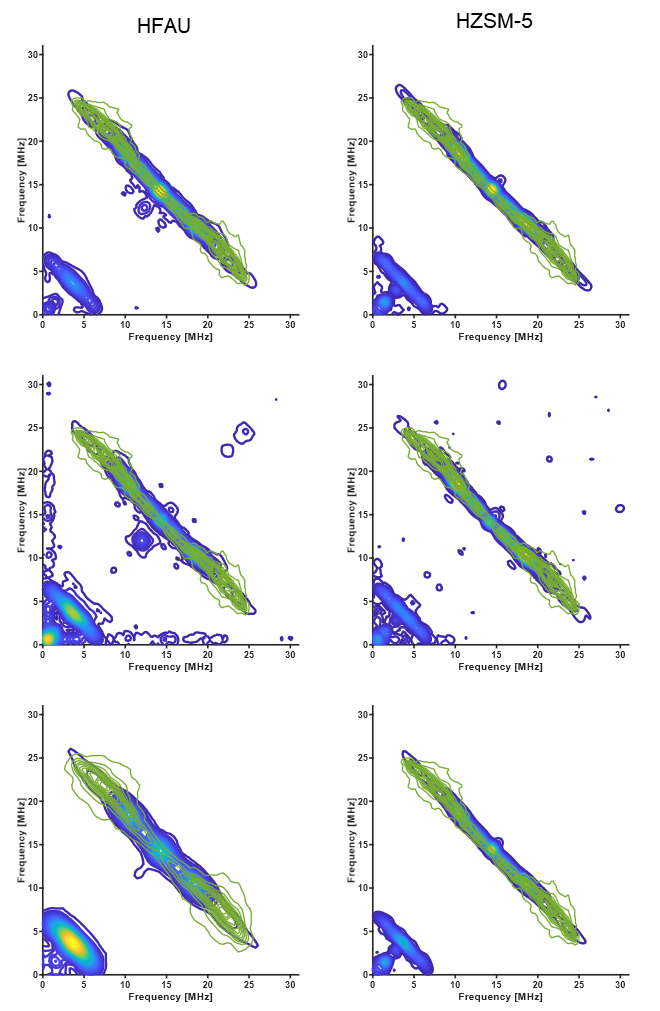
**Figure S11.** The weak interaction quadrant of the 2D HYSCORE spectra of HFAU (left) and HZSM-5 (right) from the top to the bottom after ~3 h, ~10 h, ~68 h reaction time. The green lines indicate the DFT simulated hyperfine couplings of Tetracene cation radical.


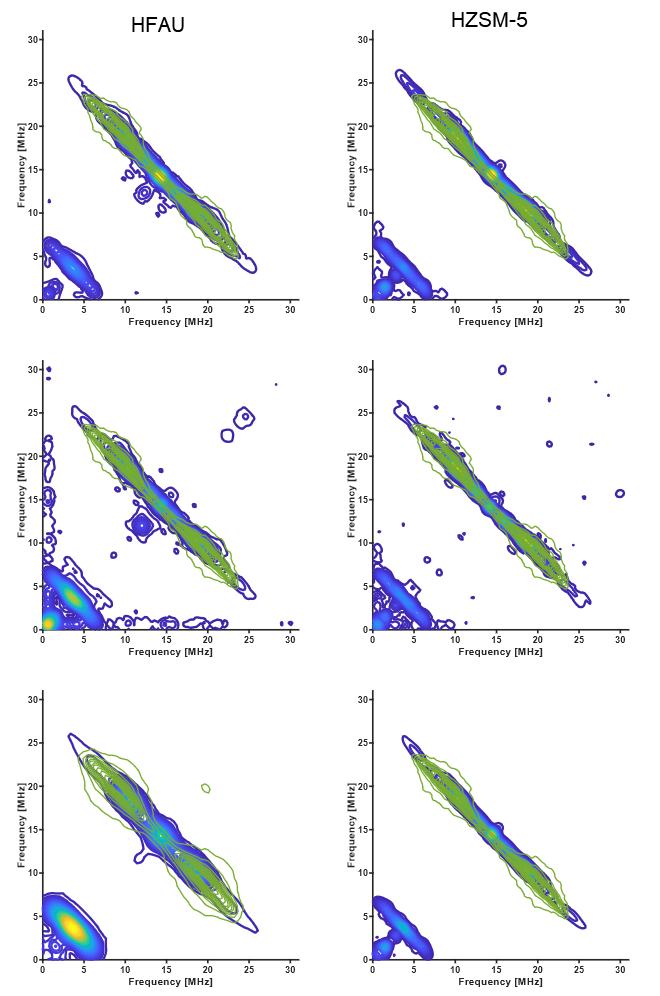
**Figure S12.** The weak interaction quadrant of the 2D HYSCORE spectra of HFAU (left) and HZSM-5 (right) from the top to the bottom after ~3 h, ~10 h, ~68 h reaction time. The green lines indicate the DFT simulated hyperfine couplings of Pentacene cation radical.


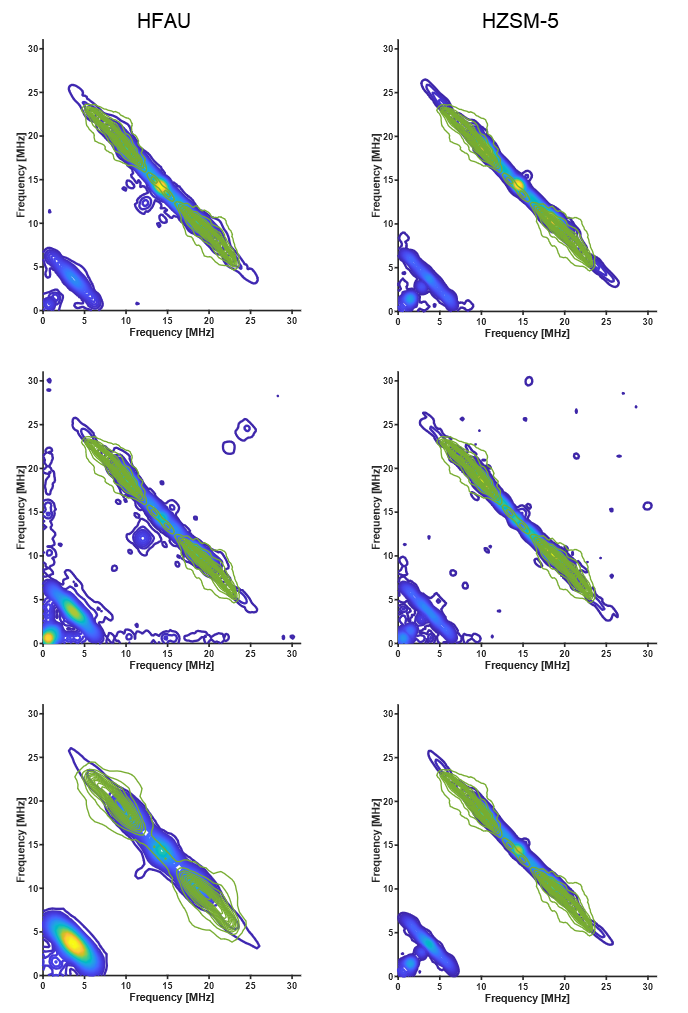
**Figure S13.** The weak interaction quadrant of the 2D HYSCORE spectra of HFAU (left) and HZSM-5 (right) from the top to the bottom after ~3 h, ~10 h, ~68 h reaction time. The green lines indicate the DFT simulated hyperfine couplings of Hexacene cation radical.
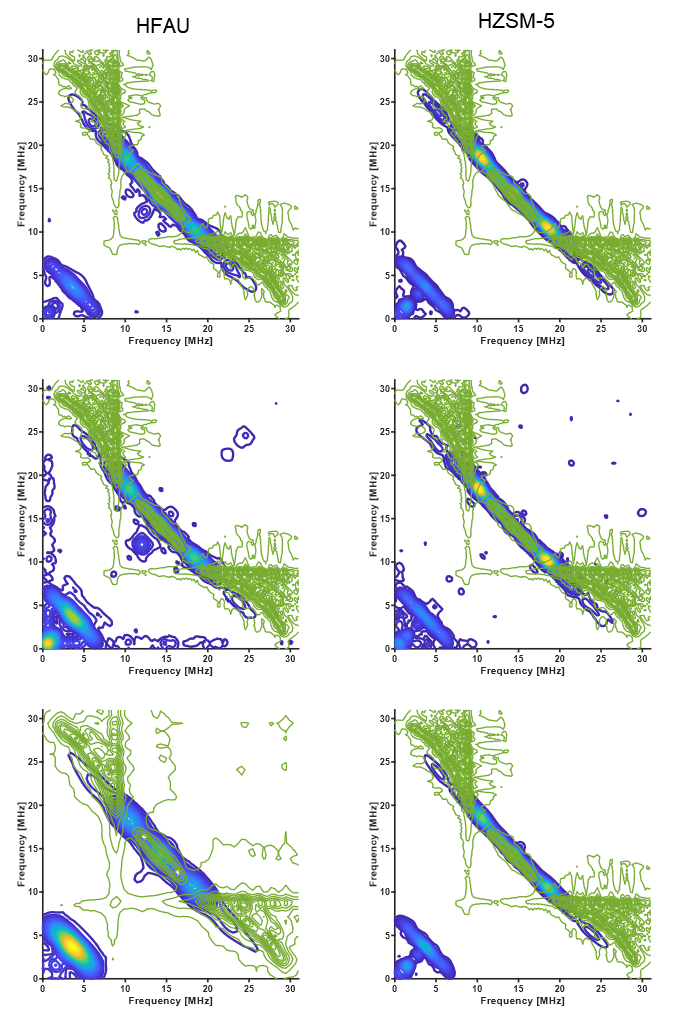
**Figure S14.** The weak interaction quadrant of the 2D HYSCORE spectra of HFAU (left) and HZSM-5 (right) from the top to the bottom after ~3 h, ~10 h, ~68 h reaction time. The green lines indicate the DFT simulated hyperfine couplings of Pentalene cation radical.**Figure S15.** The weak interaction quadrant of the 2D HYSCORE spectra of HFAU (left) and HZSM-5 (right) from the top to the bottom after ~3 h, ~10 h, ~68 h reaction time. The green lines indicate the DFT simulated hyperfine couplings of Indene cation radical.


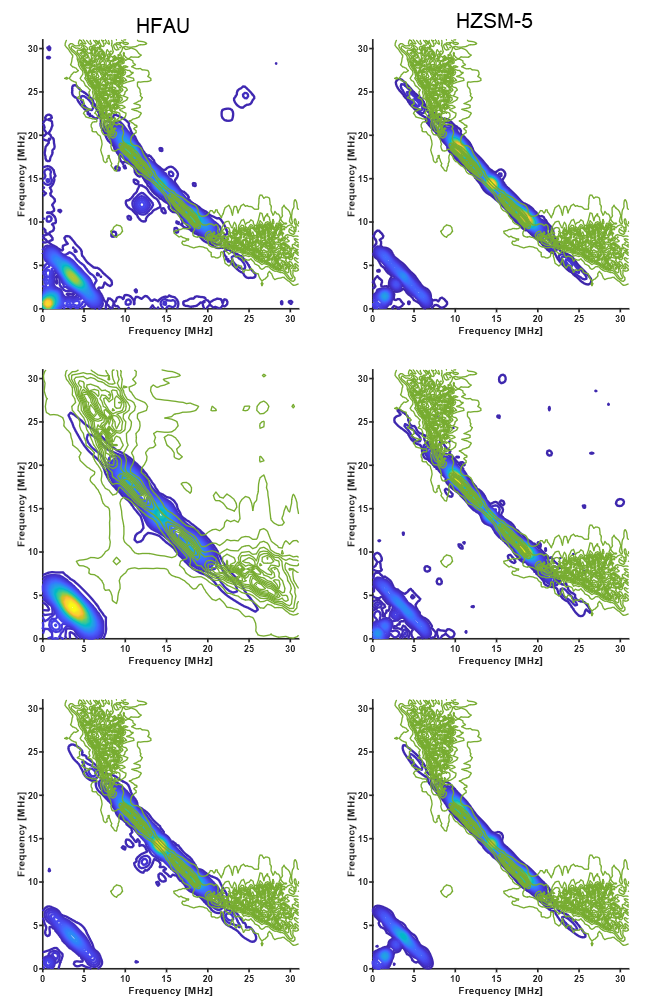

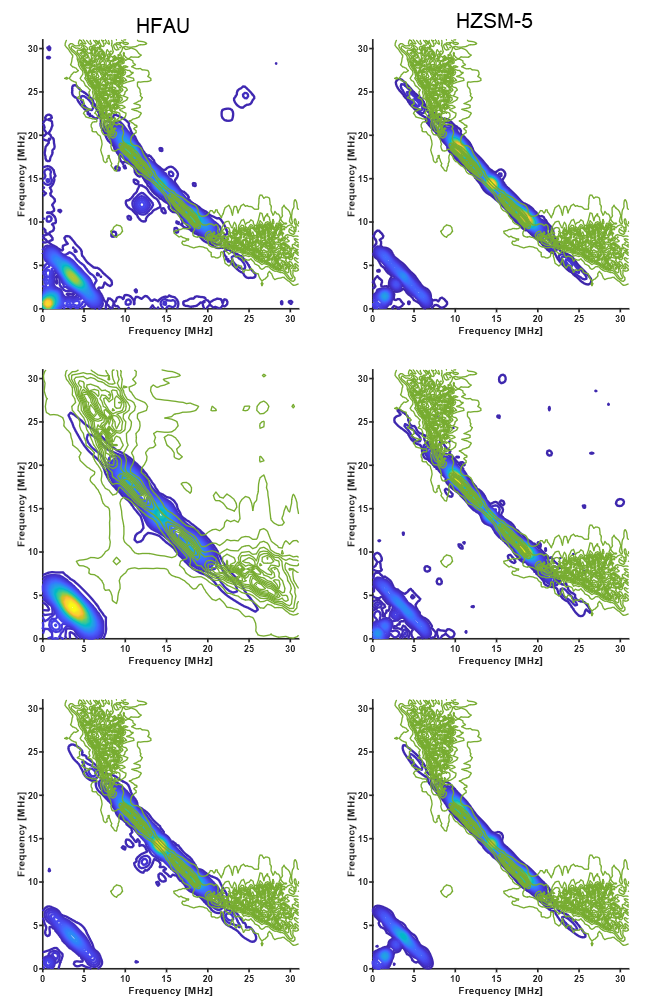

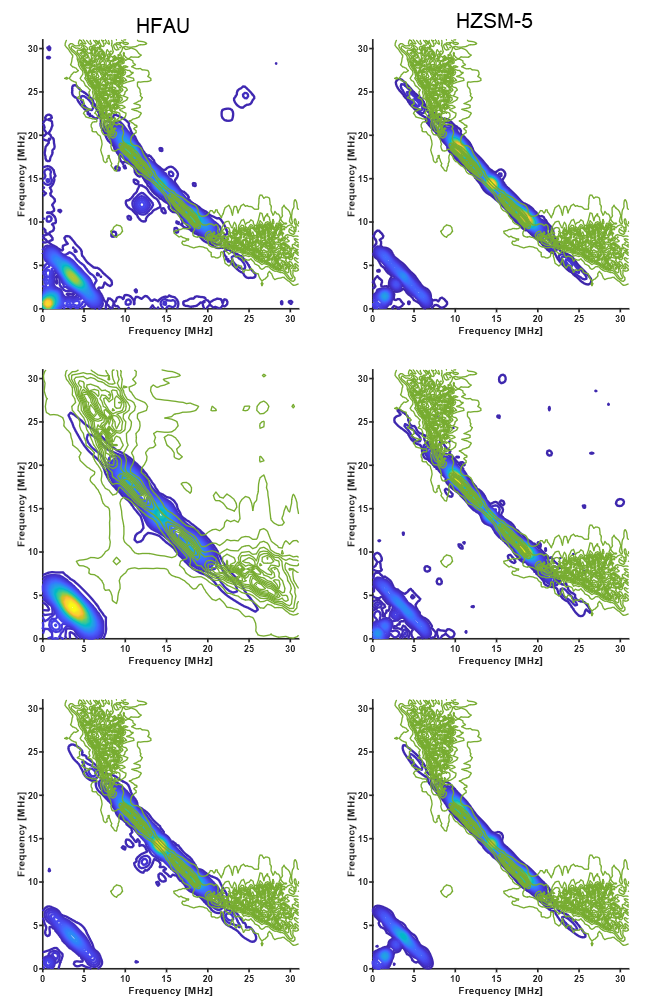


**
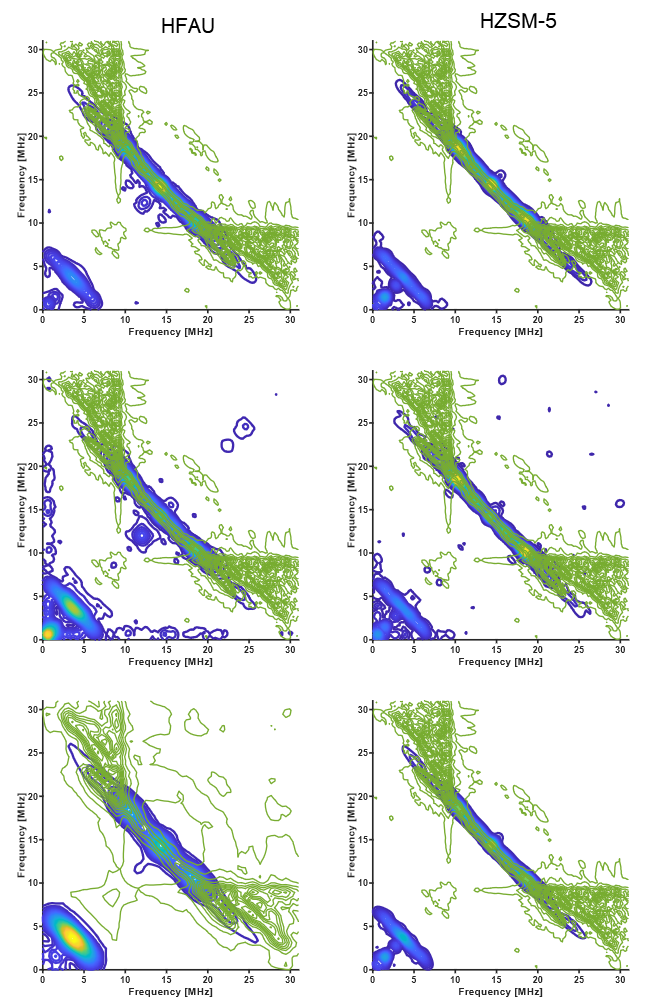
Figure S16.** The weak interaction quadrant of the 2D HYSCORE spectra of HFAU (left) and HZSM-5 (right) from the top to the bottom after ~3 h, ~10 h, ~68 h reaction time. The green lines indicate the DFT simulated hyperfine couplings of Azulene cation radical.
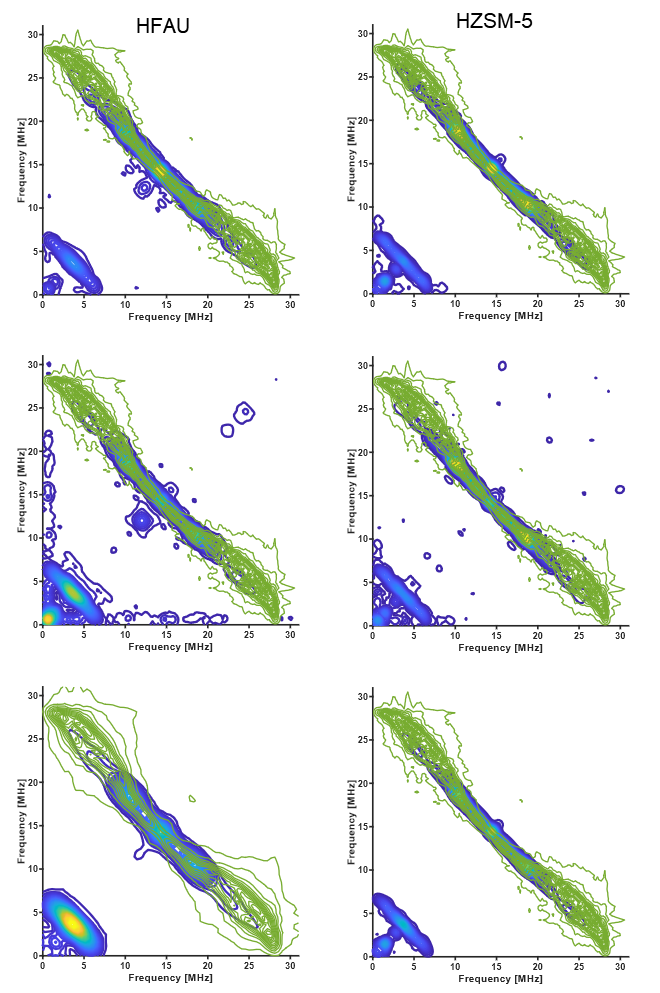
**Figure S17.** The weak interaction quadrant of the 2D HYSCORE spectra of HFAU (left) and HZSM-5 (right) from the top to the bottom after ~3 h, ~10 h, ~68 h reaction time. The green lines indicate the DFT simulated hyperfine couplings of Fluorene cation radical.


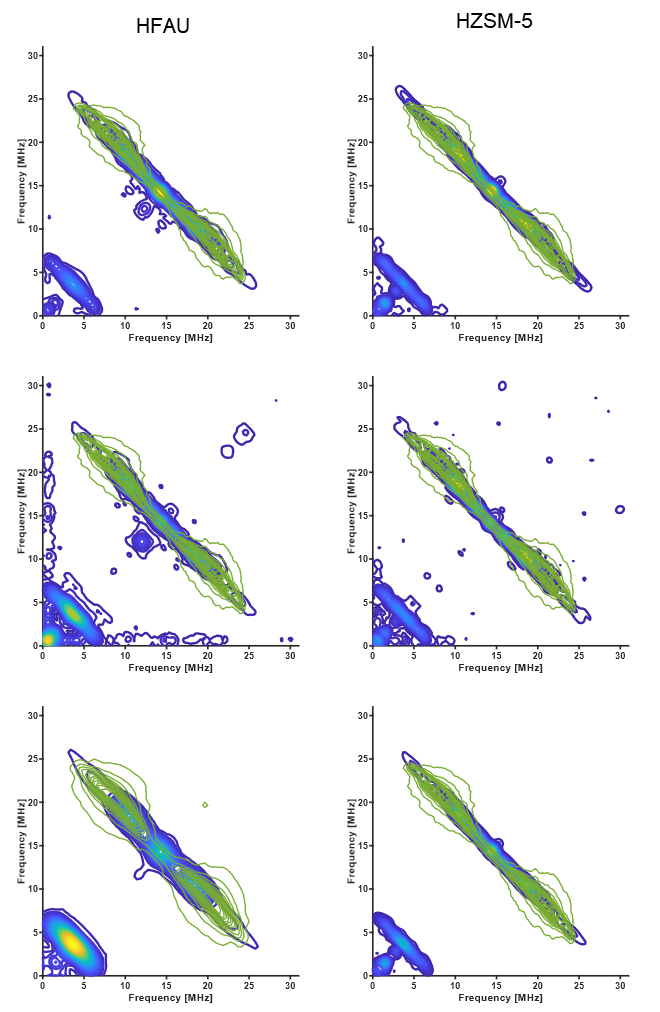


**Figure S18.** The weak interaction quadrant of the 2D HYSCORE spectra of HFAU (left) and HZSM-5 (right) from the top to the bottom after ~3 h, ~10 h, ~68 h reaction time. The green lines indicate the DFT simulated hyperfine couplings of Phenanthrene cation radical.

**
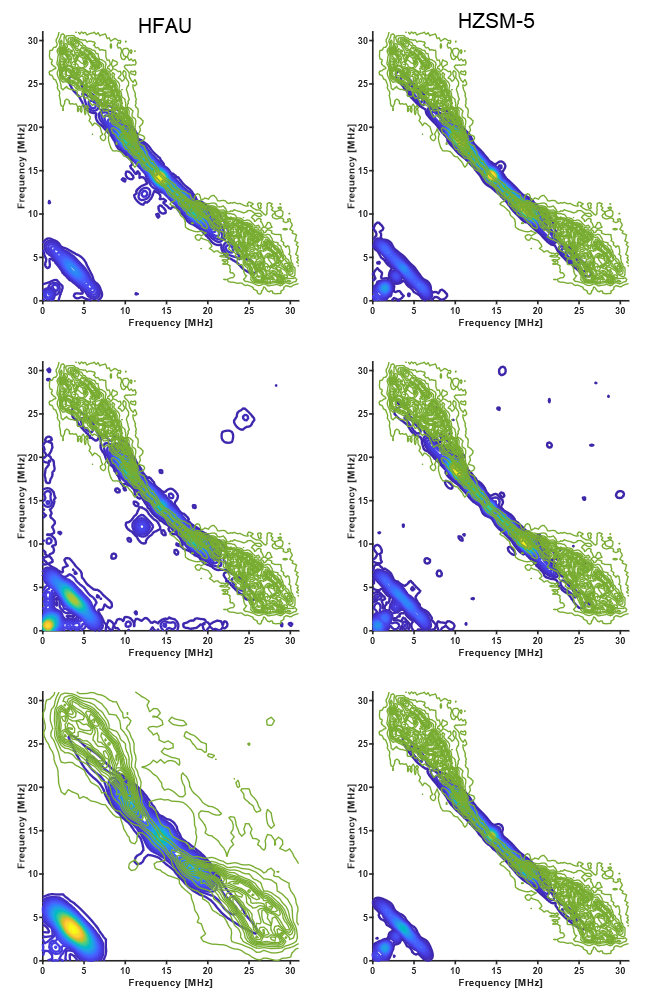
**

**Figure S19.** The weak interaction quadrant of the 2D HYSCORE spectra of HFAU (left) and HZSM-5 (right) from the top to the bottom after ~3 h, ~10 h, ~68 h reaction time. The green lines indicate the DFT simulated hyperfine couplings of Acenaphthylene cation radical.


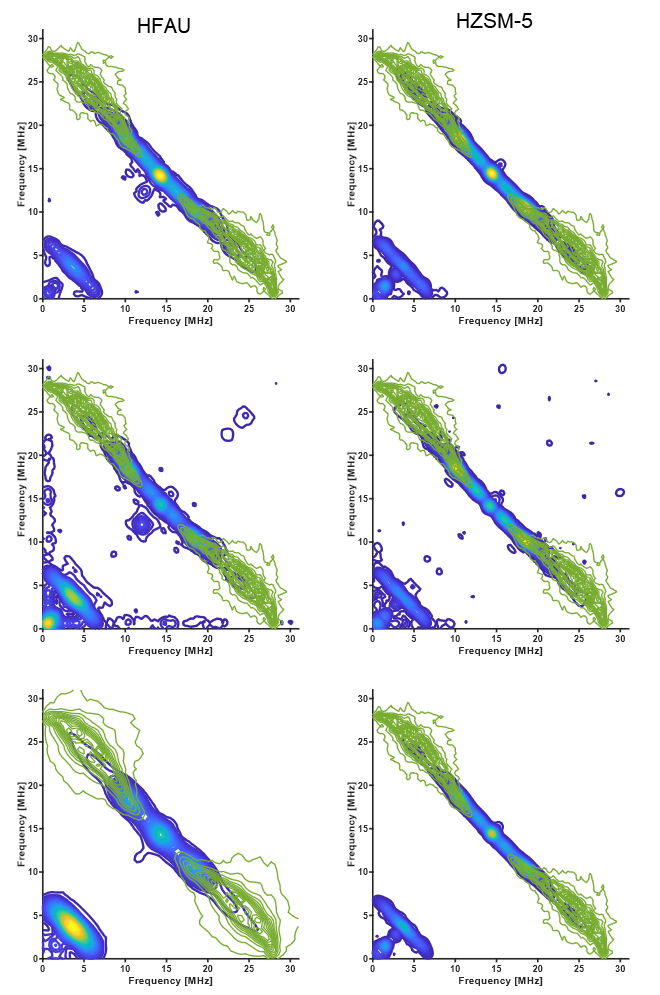
**Figure S20.** The weak interaction quadrant of the 2D HYSCORE spectra of HFAU (left) and HZSM-5 (right) from the top to the bottom after ~3 h, ~10 h, ~68 h reaction time. The green lines indicate the DFT simulated hyperfine couplings of Phenalene cation radical.**
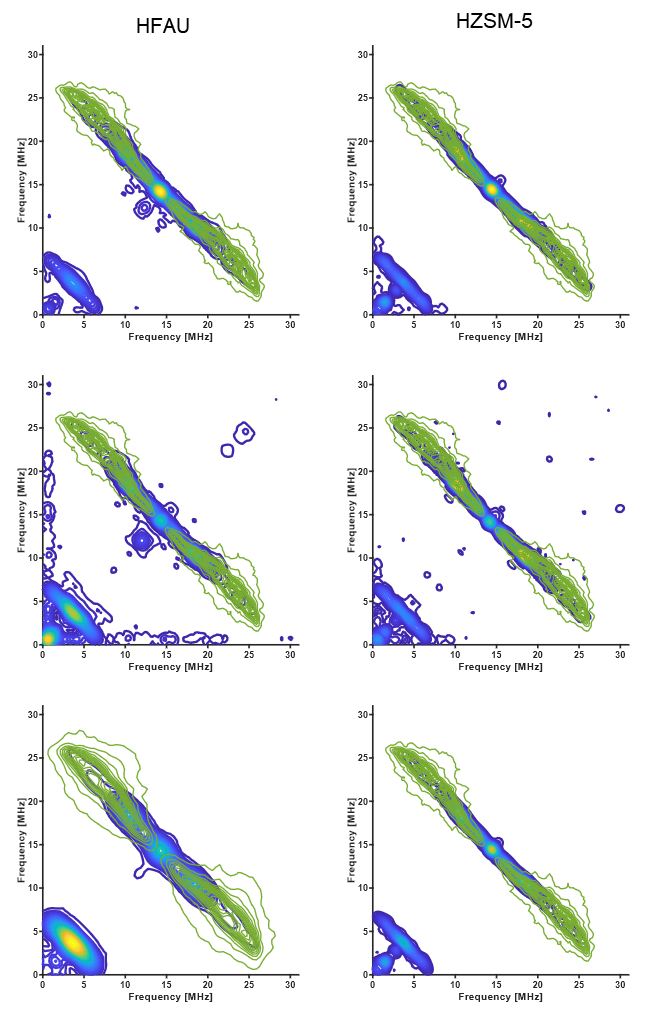
Figure S21.** The weak interaction quadrant of the 2D HYSCORE spectra of HFAU (left) and HZSM-5 (right) from the top to the bottom after ~3 h, ~10 h, ~68 h reaction time. The green lines indicate the DFT simulated hyperfine couplings of Pyrene cation radical.

**
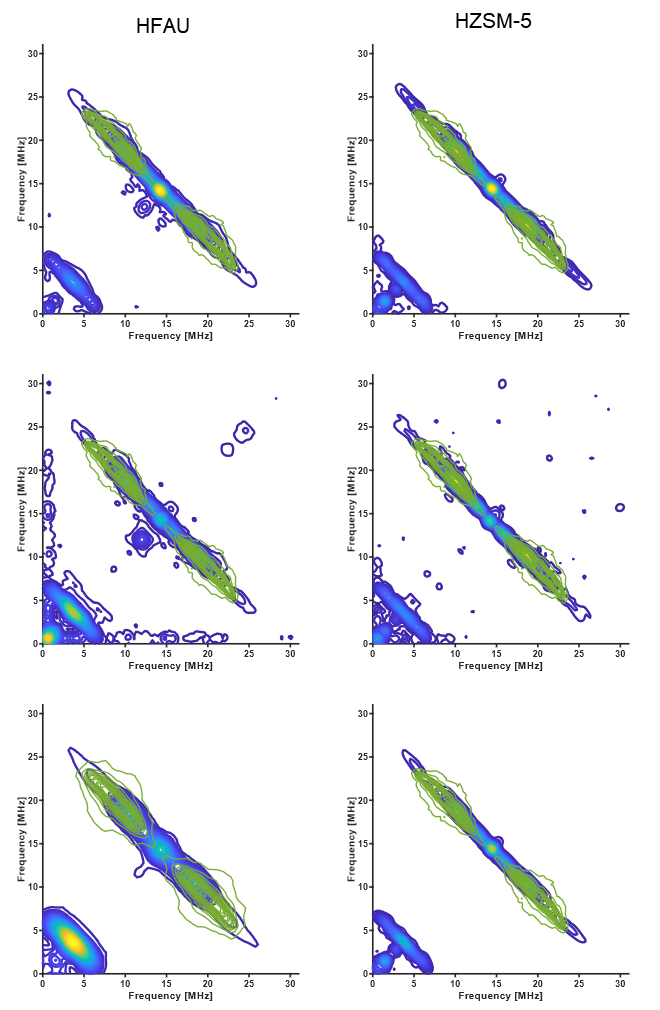
**

**Figure S22.** The weak interaction quadrant of the 2D HYSCORE spectra of HFAU (left) and HZSM-5 (right) from the top to the bottom after ~3 h, ~10 h, ~68 h reaction time. The green lines indicate the DFT simulated hyperfine couplings of Perylene cation radical.


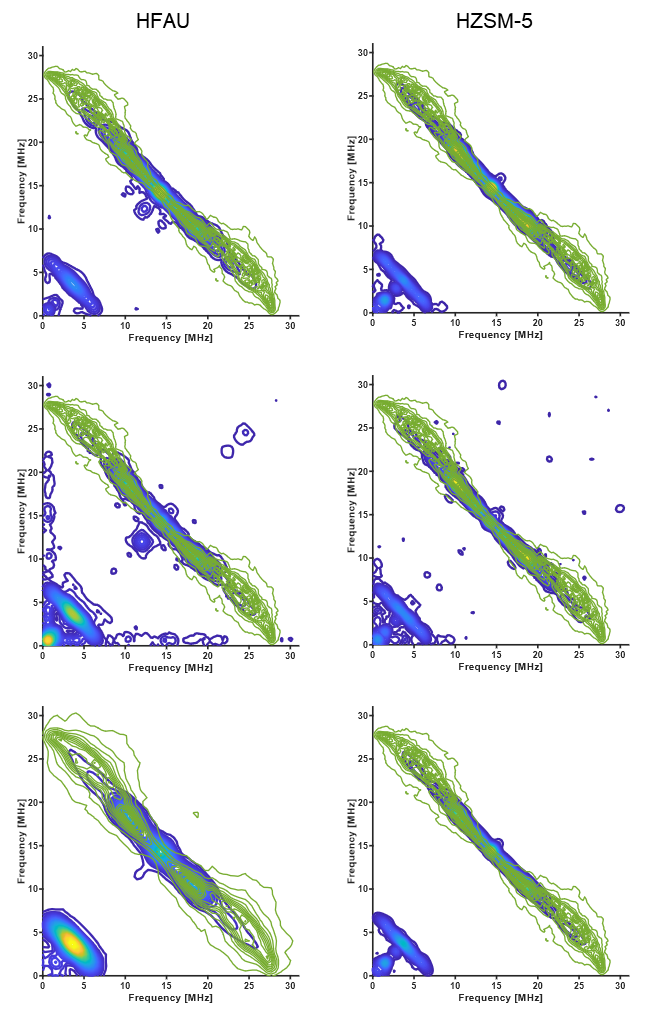
**Figure S23.** The weak interaction quadrant of the 2D HYSCORE spectra of HFAU (left) and HZSM-5 (right) from the top to the bottom after ~3 h, ~10 h, ~68 h reaction time. The green lines indicate the DFT simulated hyperfine couplings of Tetraphene cation radical.


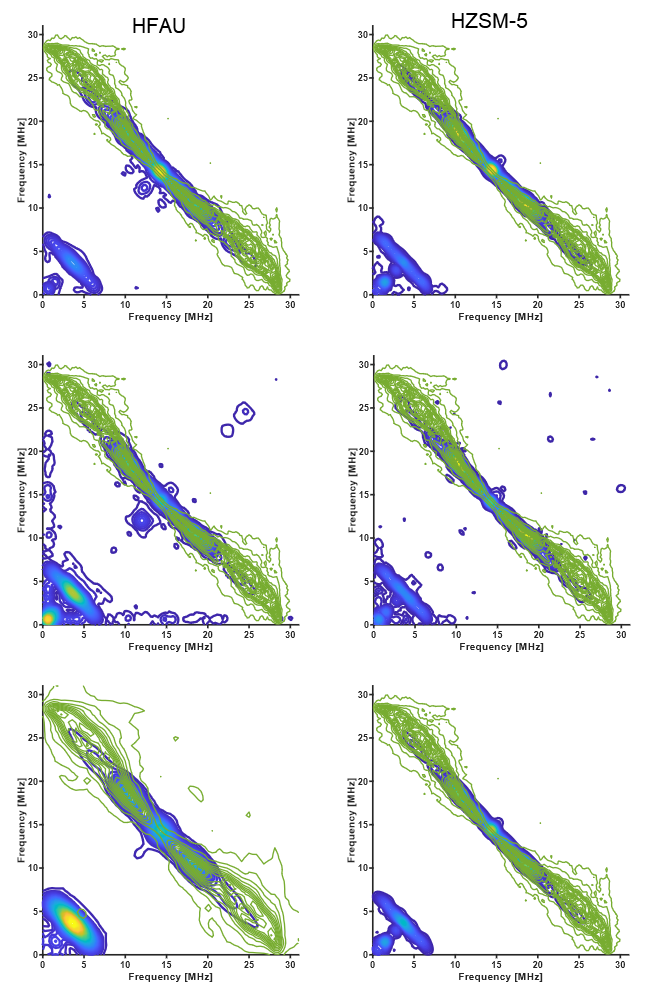


F**igure S24.** The weak interaction quadrant of the 2D HYSCORE spectra of HFAU (left) and HZSM-5 (right) from the top to the bottom after ~3 h, ~10 h, ~68 h reaction time. The green lines indicate the DFT simulated hyperfine couplings of Benzopyrene cation radical.
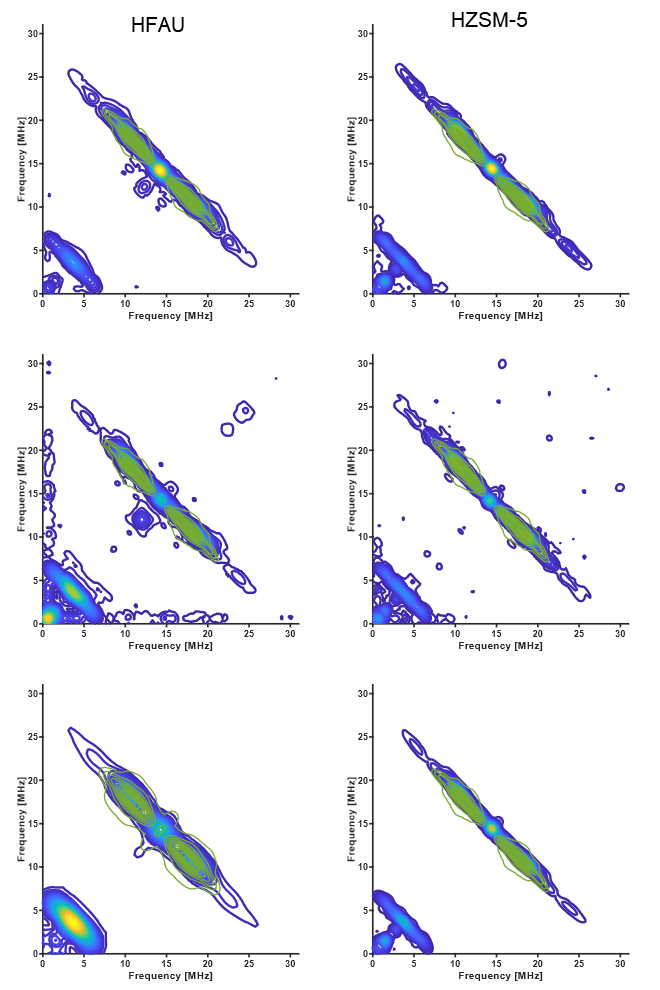
**Figure S25.** The weak interaction quadrant of the 2D HYSCORE spectra of HFAU (left) and HZSM-5 (right) from the top to the bottom after ~3 h, ~10 h, ~68 h reaction time. The green lines indicate the DFT simulated hyperfine couplings of Coronene cation radical.


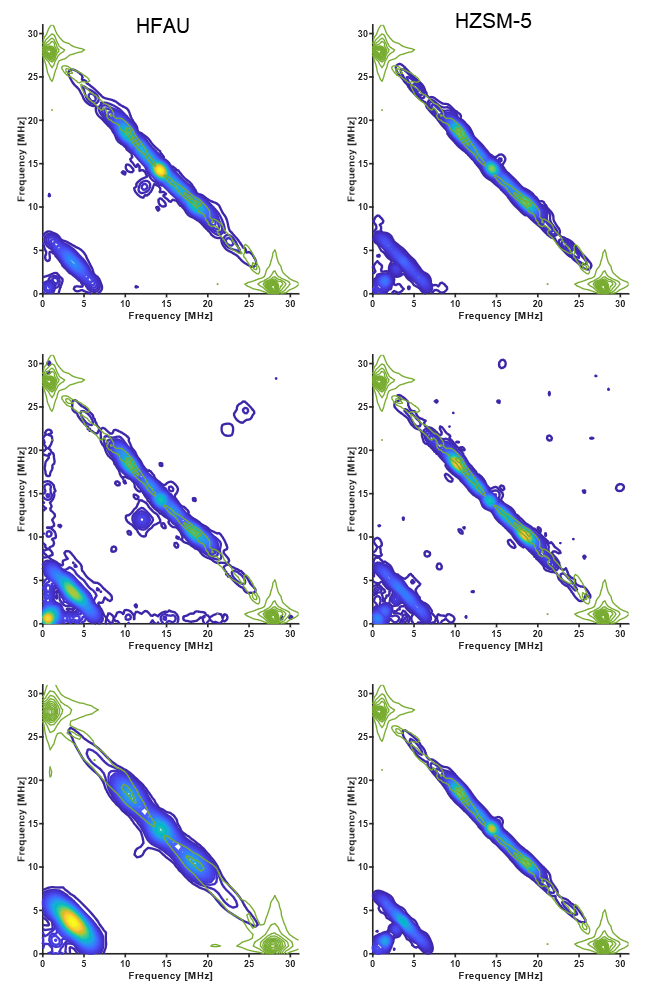
**Figure S26.** The weak interaction quadrant of the 2D HYSCORE spectra of HFAU (left) and HZSM-5 (right) from the top to the bottom after ~3 h, ~10 h, ~68 h reaction time. The green lines indicate the DFT simulated hyperfine couplings of Methylnaphthalene cation radical.


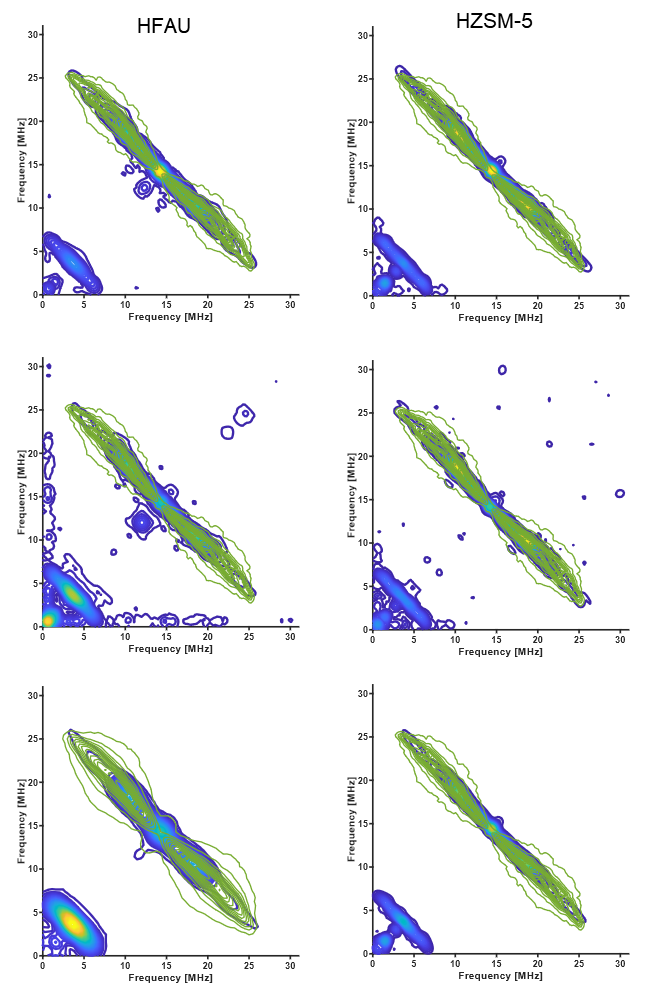
**Figure S27.** The weak interaction quadrant of the 2D HYSCORE spectra of HFAU (left) and HZSM-5 (right) from the top to the bottom after ~3 h, ~10 h, ~68 h reaction time. The green lines indicate the DFT simulated hyperfine couplings of Ethylnaphthalene cation radical.

**Figure S28.** The weak interaction quadrant of the 2D HYSCORE spectra of HFAU (left) and HZSM-5 (right) from the top to the bottom after ~3 h, ~10 h, ~68 h reaction time. The green lines indicate the DFT simulated hyperfine couplings of Dimethylnaphthalene**
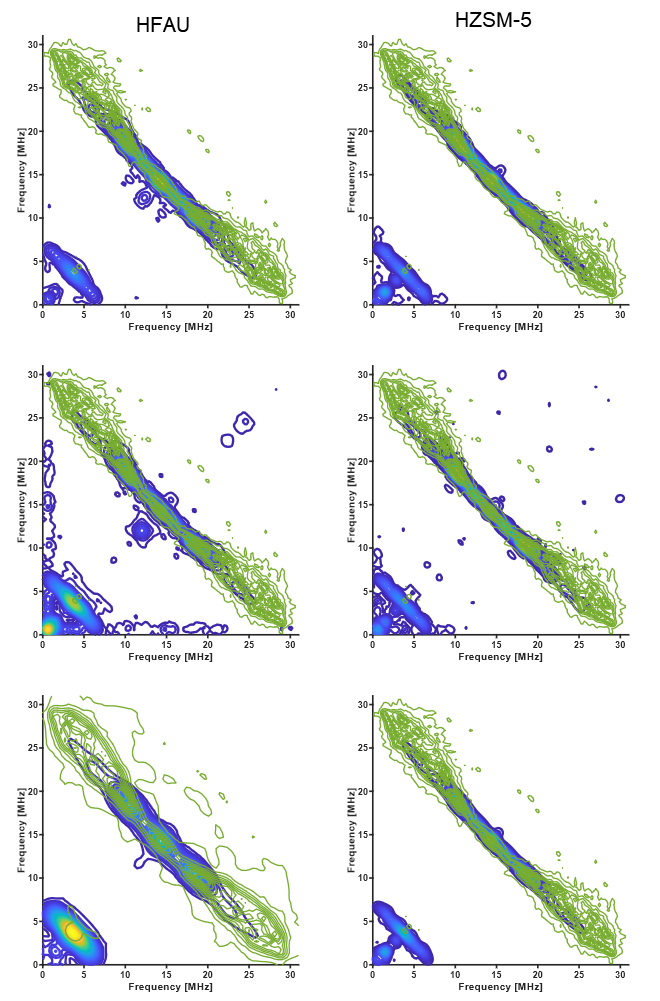
** cation radical.

**Figure S29.** The weak interaction quadrant of the 2D HYSCORE spectra of HFAU (left) and HZSM-5 (right) from the top to the bottom after ~3 h, ~10 h, and ~68 h reaction time. The green lines indicate the DFT simulated hyperfine couplings of Diethylnaphthalene**
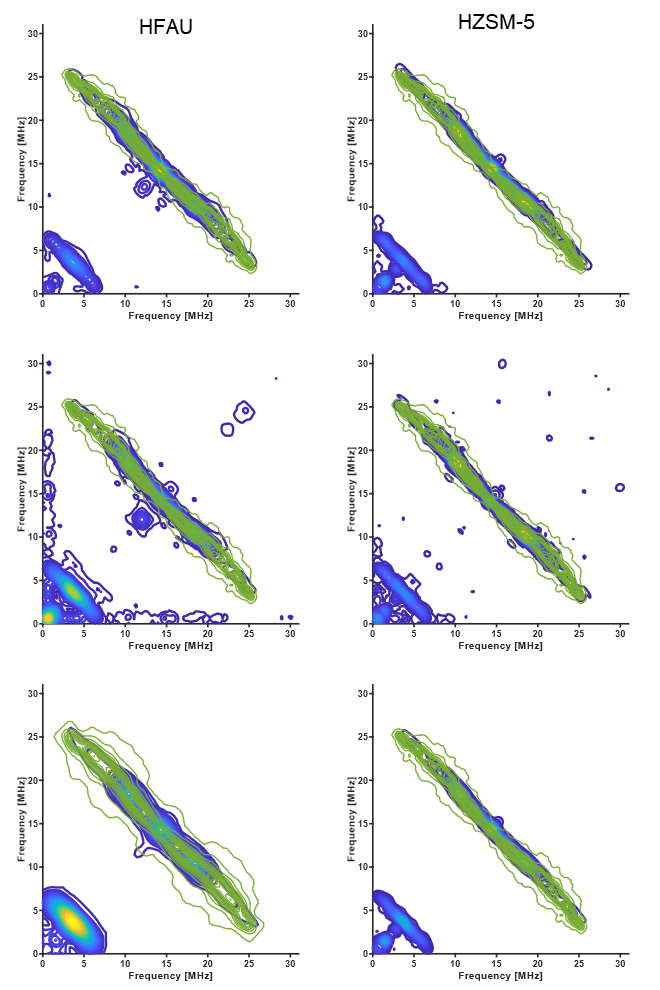
** cation radical.

**Figure S30.** The 2D HYSCORE spectra of HFAU (left) and HZSM-5 (right) from the top to the bottom after ~3 h, ~10 h reaction time. The small hyperfine couplings are highlighted with arrows.


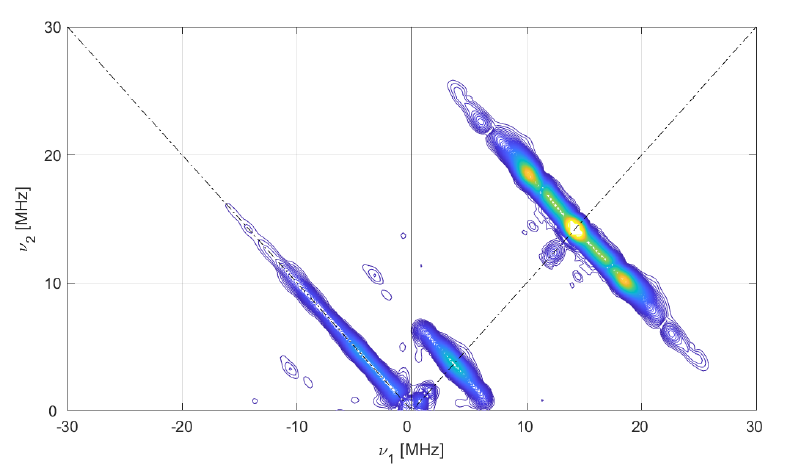

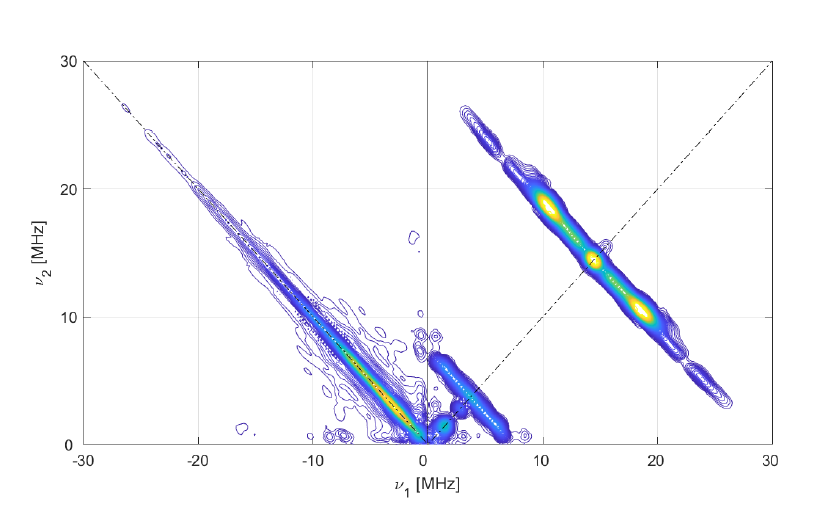

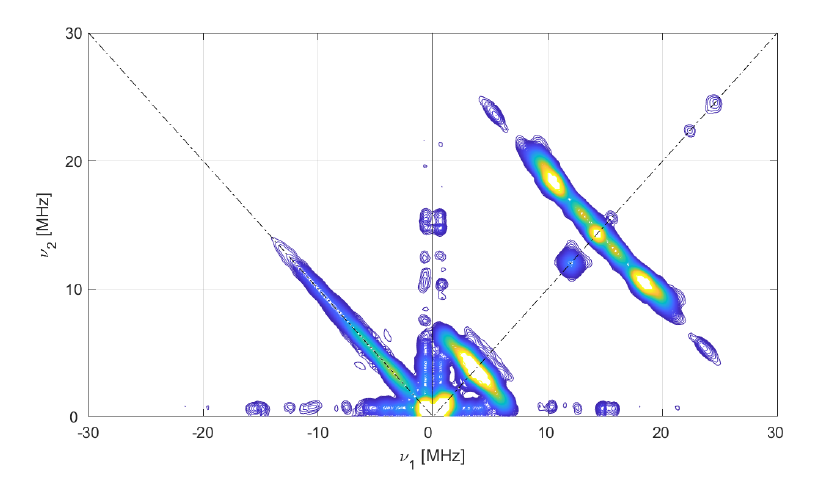

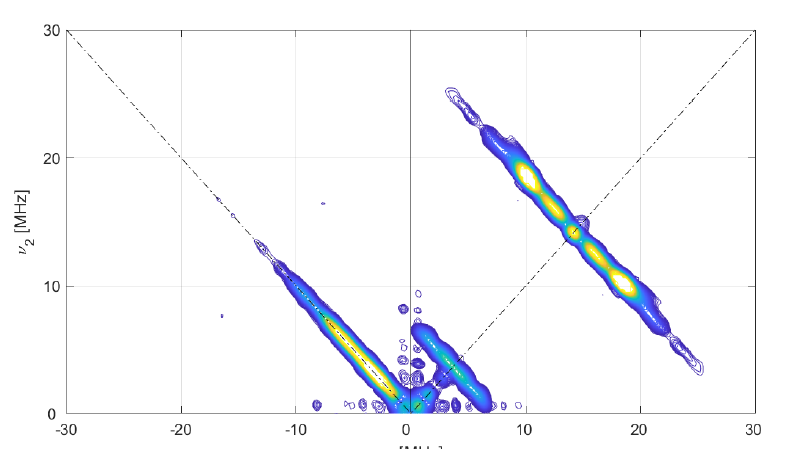

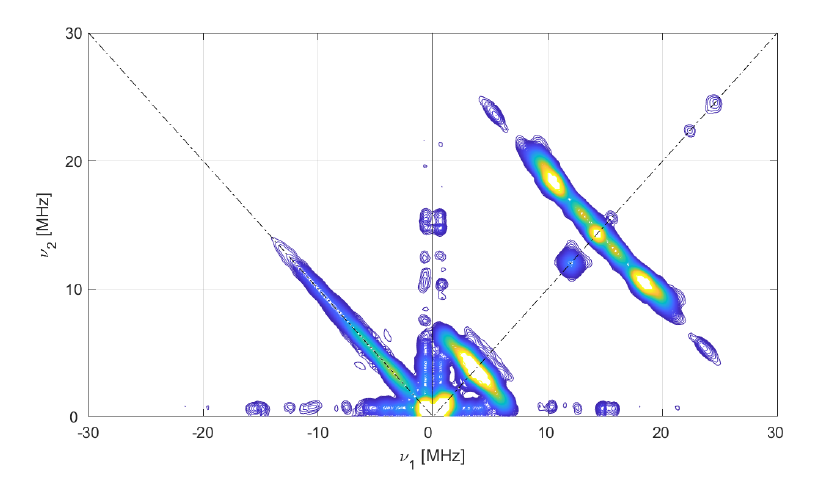


HFAU 3 h

HFAU 10 h

HZSM-5 3 h

HZSM-5 10 h

**References**

1 J. Cabana, Z. Zhang, Z. Pan, P. Kumar, X. Wu, A. Bodi, G. A. Garcia, Y. Shen, X. Xiao, H. Ma, C. Huang, C. Liu, L. Zhao, Y. Pan, Z. Zhou, J. A. van Bokhoven and P. Hemberger, *ChemSusChem*, **n/a**, 2500516.

2 Z. Pan, A. Puente-Urbina, S. R. Batool, A. Bodi, X. Wu, Z. Zhang, J. A. van Bokhoven and P. Hemberger, *Nat. Commun.*, 2023, **14**, 4512.

3 J. W. A. Fischer, F. Buttignol, A. Brenig, D. Klose, D. Ferri, V. Sushkevich, J. A. van Bokhoven and G. Jeschke, *Catal. Today*, 2023, 114503.

4 A. R. Pradhan, T.-S. Lin, W.-H. Chen, S.-J. Jong, J.-F. Wu, K.-J. Chao and S.-B. Liu, *J. Catal.*, 1999, **184**, 29–38.

5 L. Pinard, S. Hamieh, C. Canaff, F. Ferreira Madeira, I. Batonneau-Gener, S. Maury, O. Delpoux, K. Ben Tayeb, Y. Pouilloux and H. Vezin, *J. Catal.*, 2013, **299**, 284–297.

6 A. Cesarini, S. Mitchell, G. Zichittella, M. Agrachev, S. P. Schmid, G. Jeschke, Z. Pan, A. Bodi, P. Hemberger and J. Pérez-Ramírez, *Nat. Catal.*, 2022, **5**, 605–614.

7 S. Stoll and A. Schweiger, *J. Magn. Reson.*, 2006, **178**, 42–55.

8 M. Mazur, *Anal. Chim. Acta*, 2006, **561**, 1–15.

9 P. Höfer, A. Grupp, H. Nebenführ and M. Mehring, *Chem. Phys. Lett.*, 1986, **132**, 279–282.

10 L. Fábregas Ibáñez, J. Soetbeer, D. Klose, M. Tinzl, D. Hilvert and G. Jeschke, *J. Magn. Reson.*, 2019, **307**, 106576.

11 R. Gaussian, G. Trucks, H. Schlegel, G. Scuseria, M. Robb, J. Cheeseman, G. Scalmani, V. Barone, B. Mennuci, G. Petersson, H. Nakatsuji, M. Caricato, X. Li, H. Hratchian, A. Izmaylov, J. Bloino, G. Zheng, J. Sonnenberg, M. Hada and D. Fox, *Gaussian Inc Wallingford CT*.

**Author Contributions**

**Jörg W. A. Fischer:** conceptualization, data curation, formal analysis, investigation, DFT calculations, methodology, software, validation, visualization, writing – original draft preparation, writing – review & editing. **Allen Puente-Urbina:** conceptualization, methodology, project administration, supervision, validation, visualization, writing – original draft preparation, writing – review & editing. **Zeyou Pan:** conceptualization, data curation, formal analysis, investigation, methodology, software, visualization, writing – original draft preparation, writing – review & editing. **Mikhail Agrachev:** data curation, methodology, software, visualization, writing – review & editing**. Patrick Hemberger:** writing – review & editing. **Gunnar Jeschke:** funding acquisition, resources, writing – review & editing. **Jeroen A. van Bokhoven:** funding acquisition, resources, writing – review & editing.
